# Recent advances in CAR-T cell engineering

**DOI:** 10.1186/s13045-020-00910-5

**Published:** 2020-07-02

**Authors:** Ruihao Huang, Xiaoping Li, Yundi He, Wen Zhu, Lei Gao, Yao Liu, Li Gao, Qin Wen, Jiang F. Zhong, Cheng Zhang, Xi Zhang

**Affiliations:** 1 Medical Center of Hematology, Xinqiao Hospital, State Key Laboratory of Trauma, Burn and Combined Injury, Army Medical University, Chongqing, 400037 China; 2 Department of Otolaryngology, Keck School of Medicine, University of Southern California, Los Angeles, CA USA

**Keywords:** CAR-T cell therapy, Hematological malignancies, Immune therapy

## Abstract

Chimeric antigen receptor T (CAR-T) cell therapy is regarded as an effective solution for relapsed or refractory tumors, particularly for hematological malignancies. Although the initially approved anti-CD19 CAR-T therapy has produced impressive outcomes, setbacks such as high relapse rates and resistance were experienced, driving the need to discover engineered CAR-T cells that are more effective for therapeutic use. Innovations in the structure and manufacturing of CAR-T cells have resulted in significant improvements in efficacy and persistence, particularly with the development of fourth-generation CAR-T cells. Paired with an immune modifier, the use of fourth-generation and next-generation CAR-T cells will not be limited because of cytotoxic effects and will be an efficient tool for overcoming the tumor microenvironment. In this review, we summarize the recent transformations in the ectodomain, transmembrane domain, and endodomain of the CAR structure, which, together with innovative manufacturing technology and improved cell sources, improve the prospects for the future development of CAR-T cell therapy.

## Background

**Fig. 1:**
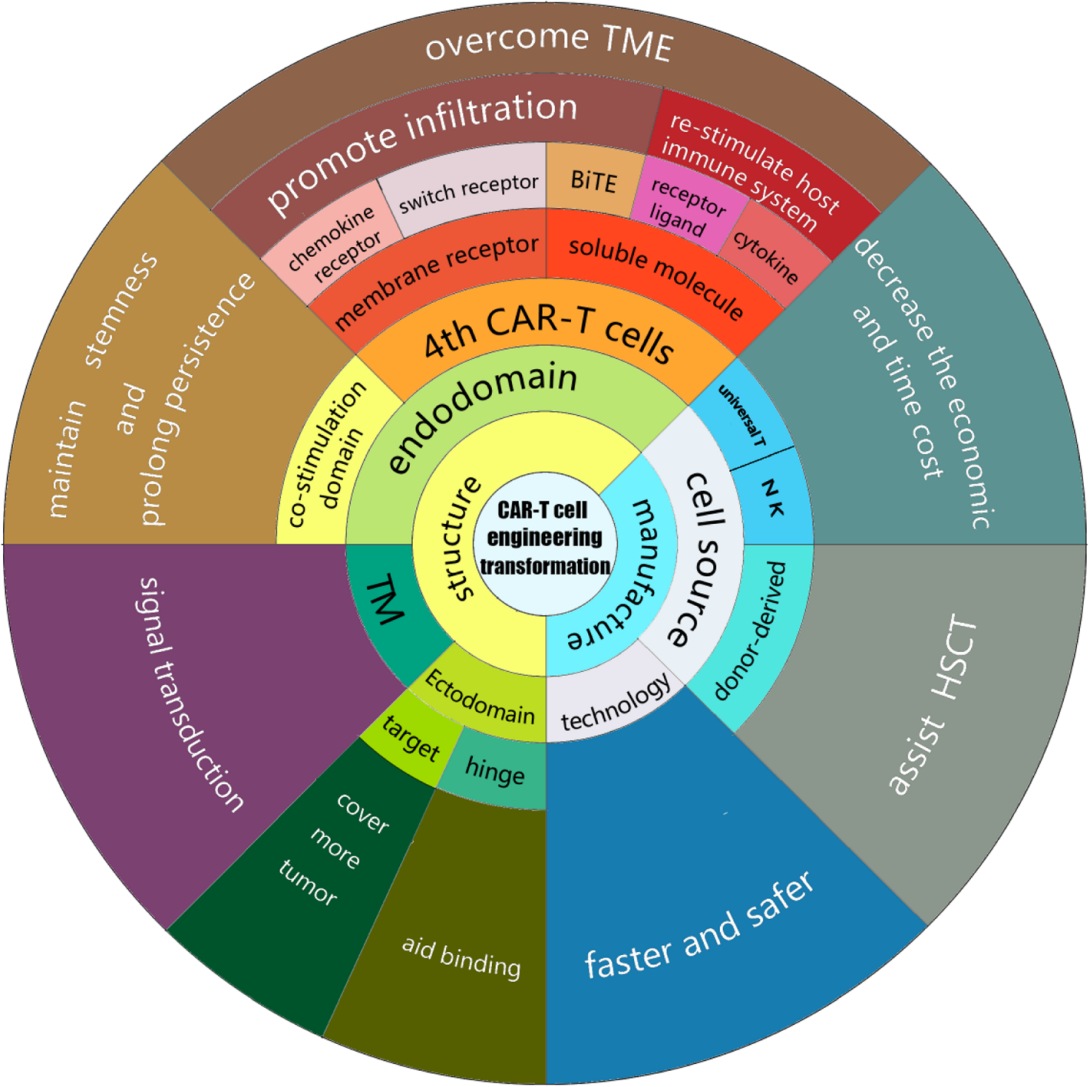
Innovation orientation and goals for transforming CAR-T cell engineering. Each cell in the inner rings with black letters represents an orientation of CAR-T cell transformation. The emerging benefits are shown in the outer rings with white letters. Clearly, in addition to improving the efficacy, the bulk of these endeavors consists of identifying the appropriate targets of the ectodomain and improving manufacturing to increase the efficacy of CAR-T cell therapy toward more diseases and ensure that is faster, safer, and more economical to satisfy more patients’ needs. After discovering the vital role of a costimulation domain choice, the importance of the transmembrane and hinge domains, as recently discovered, must be considered, as these choices affect binding and active signal transduction. As shown in this figure, substantial efforts have been devoted to develop fourth-generation and next-generation CAR-T cells. Additional areas of research were added to overcome the TME (one of the major causes of resistance to traditional CAR-T cell therapy, particularly in solid tumors). Fourth-generation and next-generation CAR-T cells are an effective tool to reconstruct the immune system of patients after the elimination of tumor cells

CAR-T cell therapy has led to a revolution in the therapy of patients with relapsed/refractory (R/R) B cell hematological malignancies [1–3]. Several phase 2 clinical trials of anti-CD19 CAR-T cells for treating R/R B cell malignancies have produced promising results. A trial of axicabtagene ciloleucel for refractory large B cell lymphoma (ZUMA-1) resulted in 82% (89/108) of the patients experiencing an overall response and 58% (63/108) achieving a complete response [4]. The administration of tisagenlecleucel (the first CAR-T drug used in the world) to adult patients with R/R diffuse large B cell lymphoma resulted in an overall response rate of 52%, with 40% of the patients achieving a complete response; the overall response rate of anti-CD19 CAR-T cells in clinical trials was greater than 80% for patients with B cell acute lymphoblastic leukemia (ALL) and non-Hodgkin’s lymphoma (NHL) [5]. In children and young adults (< 21 years old) with R/R B cell ALL, tisagenlecleucel produced an overall remission rate of 81% in the patients within 3 months, and all 75 patients who responded to treatment were negative for minimal residual disease [6]. In a clinical trial using CD19 CAR-T cells to treat chronic lymphoblastic leukemia (CLL) and small lymphoblastic leukemia (SLL), the best ORR was 82% and the best CR/CRi rate was 45.5%. Sixty-eight percent (15/22) of the patients had achieved an objective response by day 30, and 78% (7/9) of the responders remained progression-free at ≥ 9 months of post dose follow-up. For 6 patients, their responses progressed over time (3 from partial remission (PR) to complete remission (CR), 2 from stable disease to a PR, and 1 from a SD to a CR). Among the 20 people who were evaluated for MRD, most had achieved blood and/or BM MRD, and 60% (12/20) had achieved BM MRD by day 30 [7]. Despite these impressive data, the recurrence rate was high within the first year after CAR-T cell therapy. In a long-term follow-up of 101 patients with R/R diffuse large B cell lymphoma (DLBCL) who received tisagenlecleucel, 61 patients showed disease progression or died during the study. The median progression-free survival time was only 5.9 months. Some patients were unable to receive CAR-T cell therapy because of T cell apheresis failure and rapid disease progression. Moreover, “off-tumor” and nonresponse problems remained unresolved. In general, the main limitations of CAR-T cells are the limited target antigens available, vulnerability in the tumor microenvironment (TME), lack of tumor-killing ability, and low persistence. In addition, the time-consuming and expensive nature of the treatment poses challenges. Therefore, the structure of CAR-T cells, including the ectodomain, transmembrane domain, and endodomain, and the manufacturing technology and T cell sources, must be reconsidered to improve the clinical effects of CAR-T cells, including enhanced efficiency, persistence, infiltration, and anti-apoptosis ability (Fig. 1) [8–10]. The design of fourth-generation and next-generation CAR-T cells is based on this re-evaluation: these cells may provide antitumor efficacy based on mechanisms other than T cell cytotoxicity by addressing the TME and immune system reconstitution [11]. In this review, we summarize the most recent advances in CAR structure design and manufacturing and how recent progress has been used to tackle the challenges discussed above.

## The ectodomain

### Target antigen

After the successful binding target of CD19 was obtained in B cell ALL and DLBCL, studies were initiated to identify more effective binding targets. Successful targets include a B cell mature antigen (BCMA) for multiple myeloma (MM). The objective response rate of patients with MM to this target was 85% in an early trial, in which 15 of 33 patients (45%) achieved a complete response [12]. CAR-T cells engineered in this manner show exciting response rates for specific hematological malignancies, and a few phenomenal targets have been discovered, including CD20 and CD22 [13, 14] (mainly in B cell lineages). However, for many tumors, no suitable known target is currently available for CAR recognition and binding, resulting in modest outcomes in clinical trials. An ideal target should be highly specific for the tumor and have wide tumor coverage to ensure both safety and effective tumor clearance, and stability also plays a key role in the duration of the response [15]. However, few antigens meet all three requirements, and methods have been proposed to cover the shortage of targets.

One of the major challenges in identifying targets to ensure safety is avoiding “on-target off-tumor” effects: the nonspecific expression of a target antigen on healthy cells stimulates CAR-T cells and causes damage to healthy tissues, which may pose a threat to the patient’s life. For example, in malignancies of the myeloid lineage, antigens such as CD123 or CD33 are challenging targets to use because they are also expressed in vital bone marrow stem cells; therefore, although treatments targeting these antigens eliminate the tumor cells, the stem cells in the bone marrow are also killed, leading to myelosuppression. This intolerable side effect makes strategies targeting CD33 or CD123 a double-edged sword; therefore, two promising approaches have been proposed to take full advantage of this type of tumor-associated antigen (TAA). The first approach is based on an RNA carrier that expresses a CAR that avoids long-term myelosuppression. Preclinical experiments showed promising results, but a clinical trial showed no observable myelosuppression or antitumor effects on a cohort of 7 patients [16–18]. The other approach is based on protecting the bone marrow stem cells using hematopoietic stem cell transplantation (HSCT) to eliminate the anti-CD123/CD33 CAR-T cells. Using this approach, leukemia cells are eliminated, but the CD33-silent hematopoietic stem cells are protected from apoptosis induced by the CAR-T cells. The feasibility of this approach has also been reported in preclinical studies [19], but its effectiveness remains to be verified in clinical trials. We undoubtedly hope to identify a more specific TAA to serve as the binding domain for a CAR that is able to kill tumor cells while minimizing damage to normal cells, but in practice, the discovery of this TAA remains the main challenge to the extensive use of CAR-T cells as a treatment for the majority of tumors and must be resolved in the coming years.

The interaction of a CAR and its binding target determines the tumor-killing effect and the proliferation of the constructed CAR-T cells, which depend on the density of the antigen, the binding affinity, and the binding strength of the antigen. In other words, the stability of the target antigen determines the persistence of the response to some extent. Malignant cells may downregulate the target antigen to avoid CAR-T cell-induced death, which is one of the major drivers of antigen-positive relapse [20]. One promising strategy to solve this complex problem involves lengthening CAR-T cell persistence by re-engineering the intracellular region, which is discussed later in the section describing the endodomain. Another mechanism by which tumors evade treatment is through their heterogeneity, which is particularly effective when the coverage of the target antigen is limited and is the main reason for antigen-negative relapse after CAR-T cell therapy because unpredictable mutations can cause a loss of the target antigen. Therefore, as explained above, the chosen antigen must be universally expressed in the tumor. A small number of residual tumor cells can cause the disease to relapse with full resistance to the CAR-T cells targeting the specific epitope. Fortunately, this type of relapse is overcome using dual-target or combined-target CAR-T cells [20]. The primary outcome of dual-target CAR-T cell therapy in B cell malignancies is summarized in Table 1, which indicates the responses to suitable dose levels compared to the responses observed after treatment with single-target CAR-T cells. Notably, in the clinical trials cited, antigen-negative relapse was rare, suggesting that dual CAR-T cell therapy is a promising method to prevent the relapse caused by antigen escape. As shown in the table, several methods are currently available to produce a dual-target CAR-T cell involving a bispecific CAR and two different CARs carried by one or two carriers, but more clinical data are needed to select the best method to achieve dual-target CAR-T cell therapy. In addition to B cell malignancies, trials of dual anti-CD19 plus BCMA CAR-T cells for MM have also delivered promising results, with 20 (95%) of 21 patients achieving an overall response, including nine (43%) with a stringent complete remission (CR), three (14%) with a CR, five (24%) with very good partial remission (VGPR), and three (14%) with PR [31]. Another clinical trial of CD38 plus BCMA CAR-T cells for patients with R/R MM resulted in 14 (87.5%) patients achieving an overall response, 8 (50%) achieving sCR, 2 (12.5%) achieving VGPR, 4 (25.00%) achieving PR, and 14 (87.5%) achieving a bone marrow MRD-negative status. The longest duration of a sCR exceeded 51 weeks, and 5 of 8 patients (62.5%) maintained sCR, 2 progressed to VGPR, and 1 to a PR. The PFS rate at 9 months was 75% [32]. Furthermore, the combined-target CD22 CAR-T cells have been proven capable of treating patients who are resistant to CD19 CAR-T cells [14]. Nine of ten patients who had previously received CD19-directed immunotherapy achieved CR, including all five patients who had enrolled with CD19dim- or CD19-negative B-ALL and one patient who was refractory to both CD19 CAR T cell and blinatumomab therapies. Although no side-by-side comparison of single-target CAR-T cells and dual-target CAR-T cells are available to compare their levels of efficacy, multitarget CAR-T cell therapy might improve clinical outcomes by decreasing the relapse rate. However, for dual CAR-T cell therapy, managing the target epitopes to include molecules other than those in B cell lineage diseases is the next challenge to minimize the possible damage caused by the “on-target off-tumor” effect and prolong persistence to avoid antigen-positive relapse. In contrast to patients carrying only the universal target antigen, a fraction of patients carrying a mutation that causes the expression of a specific antigen that is not normally expressed in the tumor-origin tissue, such as CD7 or Lewis Y on AML cells, is able to receive CAR-T cell therapy that is relatively highly specific and that does not harm the normal myeloid cells; for example, Lewis Y is an antigen expressed on T cells but not on normal myeloid cells. The first clinical trial of anti-Lewis Y CAR-T cells for AML, in which 5 patients were treated, revealed that only one patient achieved a transient CR, while two achieved PR and the other two maintained a stable disease throughout the trial. However, all the subjects died within 1 year because of disease progression [33]. Based on these results, a satisfactory therapeutic effect may difficult to achieve when treatments rely solely on these rare target epitopes, and combining these epitopes with other widely covered targets may be a more promising strategy.

**Table 1:** The preliminary results of dual and combined CAR-T cell therapy for B cell malignancies

Researchers	Condition	Target	Design	Co-stimulation domain	Enrolled pts	Primary outcome	Long-term follow-up
Hossain et al. [21]	B cell malignancies	CD 19 and CD 22	One CAR with two binding sites	4-1BB	5 DLBCL pts, 2 ALL pts	2/6 pts achieved CR; 3/6 pts achieved PR; 1/6 pts had PD	Two pts remained CR at two and 3 months, 2 pts remained PR and the other one died of PD.
Shah et al. [22]	B-NHL	CD 19 and CD 20	One CAR with two binding sites	4-1BB	3 MCL pts, 2 DLBCL pts, and 1 CLL pts	2/6 pts achieved C R; 2/6 pts achived PR, and 2/6 pts had PD	2 pts remained in CR at 3 and 9 months
Amrolia et al. [23]	B-ALL	CD 19 and CD 22	One vector encoding two CARs	4-1BB for CD 19, OX4O for CD 22	9 CAR pediatric pts	All 9 pts achieved MRD-CR	3 pts relapsed within 1 year after treatment.
Ardeshna et al. [24]	D LBCL				11 adult pts	The lowest dose: 2/7 pts achieved CR, 2/7 pts achived PR The higher dose: 2/4 pts achieved CR	NA
Schult et al. [25]	B-ALL	CD 19 and CD 22	One CAR with two binding sites	4-1BB	10 pediatric pts and 9 adult pts	11/12 pts achieved CR, 1/12 pts had PD	The OSs was 92% with a median follow-up of 9.5 months
Dai et al. [26]	B-ALL	CD 19 and CD 22	One CAR with two binding sites	4-1BB	6 adult pts	All 6 pts achieved CR	3 relapse at 3 monthd, 5 months, and 10 months after treatment
Yang et al. [27]	B-ALL	CD 19 and CD 22	Two vectors encoding two CAR	4-1bb and extra PD-L1 for CD 22	5 children and 10 adults	All 15 pts achieved CR, 14 of them achieved MRD	11 pts bridged allo-HSCT remained in remission state with a median follow-up of 133 days. 2 pts without allo-HSCT relapsed on day 240 and day 105 after treatment
Yang et al. [28]	B-ALL	CD 19 and CD 22	One CAR with two binding sites	4-1BB	4 adults and 13 pediatrics pts	The low dose: 3/4 pts had non-response and 1/4 achieved MRD + CR.The medium dose: all 7 pts achieved CR, 6/7 pts had MRD-CR	No one relapsed with a median follow-up time of 60 days
Gardner et al. [29]	B-ALL	CD 19 and CD 22	Two vectors encoding two CARS	4-1BB	7 young adult or pediatric pts	5/7 pts achieved CR, 4/7 of them achieved MRD–	NA
Wang et al. [30]	B-ALL	CD 19 and CD 22	Manufacture and infuse separately	CD 28 and 4-1BB for both CAR	51 adult pts	48/50 pts achieved MRD-CR, 2/50 pts achieved PR	The median PFS was 13.6 months,The median O S was 31.0 months
	B-NHL				38 adult pts	18/36 pts achieved CR, 8/36 pts achieved PR	The median PFS was 9.9 months ,The median O S was 18.0 months

To date, the identification of effective targets remains a slow procedure, but with the development of sequencing techniques, we remain optimistic that an effective target for each type of tumor will be discovered in the future.

The development of single-chain variable fragment (scFv) CARs was also revolutionary. Currently, most available CAR-T cell therapies have adapted murine CARs, which are recognized by the immune system as foreign antigens; this source of antigen is also the main reason for the short persistence of CAR-T cells. Humanized CAR-T therapy has been proposed to minimize the influence of murine CARs and prolong the persistence of CAR-T cells. In a clinical trial of patients with B-ALL, after 14 days of CAR-T cell infusion, 19/23 (82.6%) patients achieved CR/CRi, 12/23 (52.2%) patients achieved CR, and 18 (78.3%) patients were MRD-negative. The other 4 patients were evaluated as NR. One of the NR patients achieved CR after 2 months of infusion with anti-CD22 CAR-T cells [34]. Another similar clinical trial was able to evaluate the response of all 10 patients with R/R ALL recruited for the study, and all achieved CR; six remained in a CR state for more than 18 months without further treatment. Long-term persistence of humanized CAR-T cells was observed in most of the patients [35]. Although the primary outcome of the humanized CAR-T cells showed a similar response rate to murine CAR-T cells, the humanized CAR-T cells appeared to provide greater long-term benefits than the murine CAR-T cells by persisting longer. Furthermore, in another clinical trial, 4 of 5 patients with B-ALL who relapsed after treatment with murine CAR-T cells or who had no initial response to murine CAR-T cells successfully achieved CR after being infused with humanized CAR-T cells [36]. The human-derived CAR structure may be an efficient and promising method to prolong the persistence of CAR-T cells, and the longer persistence indeed improves the duration of the response.

As explained above, multiple-target CAR-T cell therapy may improve the antitumor efficiency and prevent the relapse caused by antigen loss. The CAR structure is separated and the sequence that encodes only the inner and connecting parts is implanted into T cells to increase the coverage provided by the manufactured T cells. Then, the binding sequence is injected into the patients, and a specific CAR target that depends on the molecule injected into the patients and achieves universal coverage is produced. The potential for universal CARs, which can shift the binding epitope through biotin/avidin and leucine zippers, has also become a topic of significant interest. Lohmueller et al. created AT-CARs using an affinity-enhanced monomeric streptavidin 2 (mSA2) biotin-binding domain that, when expressed on T cells, targets cancer cells coated with biotinylated antibodies, while Cho et al. presented a split, universal, and programmable (SUPRA) CAR system that simultaneously encompassed multiple critical “upgrades,” such as the ability to switch targets without re-engineering the T cells, fine-tune the T cell activation strength, and sense and logically respond to multiple antigens [37, 38]. However, antigen shifting requires the persistent activation of CAR-T cells. In addition, a bispecific T cell engager with activated T cells has proven effective in some patients with advanced tumors, and these treatments are “off the shelf” and inexpensive. Further improvements are needed in the inner part of the CAR to make in vitro manufacturing worthwhile.

### Hinge domain

The ectodomain of a CAR normally has a similar structure to a monoclonal antibody (mAb), namely, the Fc region in Ig. The Fc domain mediates the antigen-antibody reaction, leading to the elimination of antibodies in a normal immune reaction. However, the Fc region exerts a negative effect on CAR-T cell persistence and function. This outcome may result from Fc-FcγR interactions between CAR-T cells and other immune cells that lead to tonic signals that accelerate T cell aging. This interaction is prevented by blocking the aging caused by the hinge and increasing the flexibility of the CAR. For example, an initially designed IgG1 Fc region has a strong affinity for FcγR, which induces tonic apoptosis-promoting signals in the CAR-T cells, for which the CH2 region is required [39]. Furthermore, knock out of the CH2 region decreased the tonic signal and prevented activation-induced T cell death. This outcome is achieved by including only the CH3 segment of an Fc region or by replacing the whole Fc region with IgG2, which has a low affinity for FcγR. This strategy avoids Fc-FcγR interaction-induced exhaustion, which prolongs the persistence of the CAR-T cells. A CD19 CAR-T cell therapy in which the Fc region was deleted also showed a high binding affinity for CD19+ malignant cells in vivo [40]. Recently launched clinical trials will clarify whether this strategy enhances the persistence and antitumor activity.

The hinge domain can also be based on linkers of membrane receptors, providing a flexible and long connection between binding sites and contributing to the binding affinity. Ectodomain linkers of CD8 alpha and CD28 were compared in CD19 anti-CAR-T cells [41], and the CD8 alpha linker generated better results, as it led to lower levels of cytokine release and less activation-induced cell death. Moreover, in a recent study, the use of NGFR as a hinge resulted in a very low affinity for FcγR and suggested that NGFR potentially represents a great traffic marker for CAR-T cell detection [42]. The flexibility of a CAR is related to its length, which contributes to the affinity of a CAR and a target antigen. In a preclinical experiment, an IgG4 CH3 hinge domain was inserted to connect the CD28 linker, and the CAR-T cells showed increased growth, migration, and CD4 subtype expansion. Researchers have not clearly determined whether the antitumor efficacy of anti-CD19 CAR-T cells is increased after the implementation of this approach. However, the addition of linkers may result in a significant increase in the activity of anti-mesothelin CAR-T cells since some target epitopes, such as CD22, require additional flexibility to achieve optimal affinity. Thus, this approach represents a promising method to improve the effects of CAR-T cells. The hinge domain was previously neglected, but the aforementioned preclinical experiment has proven that a comparable hinge domain might play a vital role in modulating the binding affinity and signal transduction, particularly for targeting dim antigens or low-affinity malignant cells.

## Transmembrane domain (TM)

The transmembrane domain connects the ectodomain and endodomain and serves as the anchor to the cell membrane. It is normally derived from a transmembrane receptor protein. The choice of the transmembrane domain influences the activating signal transmitted to the intracellular domain. In vivo comparisons indicate that the insertion of the CD8-alpha transmembrane region leads to lower levels of CD19-specific annexin V expression in T cells expressing an anti-CD19 CAR with a CD8 TM than in T cells expressing anti-CD19 CAR with a CD28 TM because the CD8-alpha TM results in lower levels of T cell activation-induced death than the CD28 TM [41]. The inhibition of AICD in this manner might prolong the circulation of the CAR-T cells. The CD8 TM has been adapted for use in most current products. However, further exploration indicates better regulatory effects of the ICOS TM on the antitumor activity and increased persistence compared to the effect and persistence induced by the CD8 TM. In a third-generation in vivo test, cells with a CD8 TM or a 4-1BB TM displayed similar, modest antitumor activities, whereas cells with an ICOS TM induced 100% tumor regression within 35 days in NSG mice. Importantly, CAR-T cells with an ICOS TM showed increased expansion and persistence in vivo compared with cells with a CD8 TM or 4-1BB TM [43]. The TM of the TNFRS family exhibits increased tumor-killing ability compared the CD8-alpha TM [44]. The primary clinical outcome of a trial of anti-CD19 CAR-T cells with TNFRS19 TM for use in patients with R/R B cell NHL achieved an overall response rate of 83% (10/12) and a complete response rate of 67% (8/12) [45, 46]. Larger clinical trials and longer observation time are needed to verify the persistence of these cells. Furthermore, changes in the length of the ectodomain and endodomain connecting regions will change the structure, leading to changes in signal transduction. According to a preclinical study, changing the length of the TM may also slow the proliferation of CAR-T cells without attenuating their tumor-killing activity, enabling patients to process inflammatory cytokines over a relatively long period [47]. Similar to the hinge domain, the advantages of the TM remain to be proven in additional clinical trials, but a reformulation of the TM might be a universal strategy for all CAR-T cell therapies and it is important for decreasing tonic signals and prolonging CAR-T cell persistence.

## The endodomain

### Development of the costimulation domain

Innovative approaches to modifying the intracellular domain have been the primary driver of revolutionary changes in CAR-T cells across generations to date. First-generation CAR-T cells with only the ITAM segment of CD3 became obsolete because they lacked persistence and a proliferation capacity. Second-generation anti-CD19 CAR-T cells showed strong antitumor efficacy in patients with B cell ALL and NHL and were formulated to contain one endodomain of a stimulation epitope, such as CD28 or 4-1BB [4–6]. Studies exploring the potential of costimulation domains are ongoing, and new costimulation domains, such as ICOS and CD27, have shown efficacy in eliminating tumor cells in preclinical experiments [48, 49]. However, these domains have consistently been shown to decrease inner tonic signaling, which causes T cell exhaustion, with 4-1BB exerting a greater effect than CD28 in preclinical and clinical trials. The CD28 costimulation domain responds quicker than the 4-1BB costimulation domain, but the population of CAR-T cells with the 4-1BB costimulation domain peaks at a higher level after a relatively slow expansion [50]. Additionally, a preclinical trial revealed that the 4-1BB costimulation domain promotes the formation of the memory Tcm subtype, while the CD28 costimulation domain promotes the formation of the Tem subtype [51], and thus, CAR-T cells with the 4-1BB costimulation domain persist longer than the CAR-T cells with the CD28 costimulation domain. In addition, compared with the CD28 CAR-T costimulaion domain, the 4-1BB CAR-T costimulation domain induces weaker tonic signaling, which means that exhaustion transpires more slowly for the CAR-T cells with the 4-IBB costimulation domain than for CAR-T cells with the CD28 costimulation domain [52–54]. However, the persistence and relapse problems associated with CAR-T therapies, in general, are unable to be solved by a single costimulation domain. Therefore, the third generation of CAR-T cells was designed with two costimulation domains. Preclinical testing showed increased persistence, proliferation, and antitumor activities. In clinical trials of the third-generation anti-CD19 CAR-T cells with 4-1BB and CD28 endodomains, the primary response rate did not show enhanced efficacy compared to the second-generation CAR-T cells in treating subjects with R/R B cell malignancies. Furthermore, for patients with MRD+ or a small burden of B cell malignancies at the time of the infusion, third-generation CAR-T cells exhibited better proliferation [55–58]. Additional preclinical experiments discovered more combinations, such as ICOS plus 4-1BB, TLR2 (Toll/interleukin-1 receptor domain of Toll-like receptor 2) plus CD28, and 4-1BB plus OX40, all of which exhibited better performance than the combination of CD28 and 4-1BB costimulation domains in vivo and in mathematical models [43, 59]. Despite these promising results, the rate of cytokine release syndrome (CRS) increased, which may be attributed to the repeated signal delivered by both costimulation domains. In summary, third-generation CAR-T cells may benefit from combining complementary costimulation domains. On the other hand, the simple addition of a costimulation domain may produce severe side effects and accelerate the aging of the CAR-T cells. Additional clinical trials are needed to assess and monitor the risk of side effects due to overactivation to realize the optimal potential of third-generation CAR-T cells.

The costimulation domains contribute substantially to the expansion and persistence of CAR-T cells for therapy. Currently, CAR-T cells with the CD28 and 4-1BB costimulation domains are the most commonly used CAR-T cell products. However, 4-1BB has several advantages compared with CD28. New costimulation domains have been discovered and tested, but the tonic signaling problem remains unresolved. The use of more than one costimulation domain may increase the tumor-killing activity and persistence of CAR-T cells, but a poor match might lead to increased tonic signaling, which would result in more severe side effects and faster T cell exhaustion.

### Fourth-generation and next-generation CAR-T cells

**Fig. 2:**
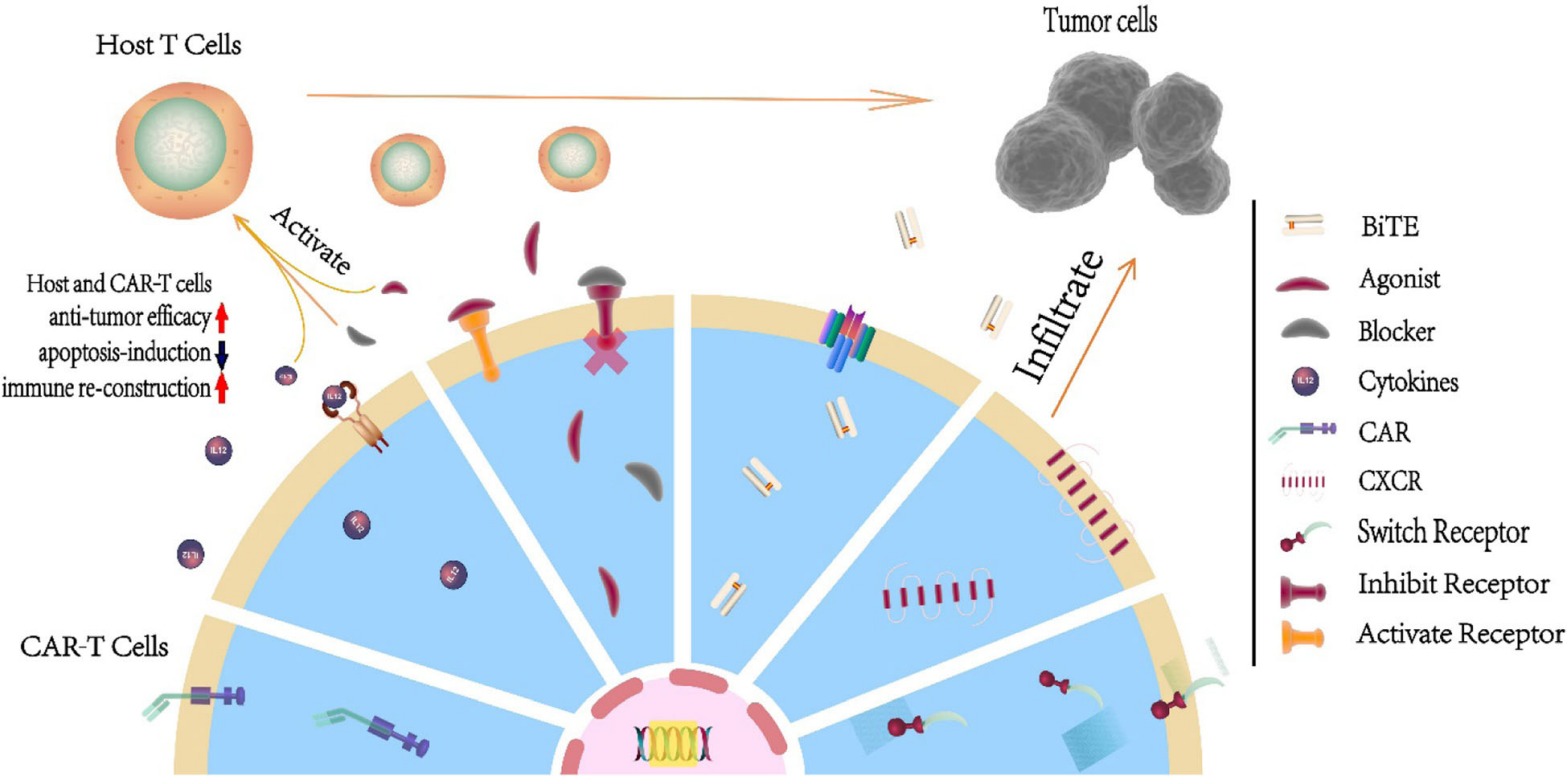
The antitumor mechanisms of fourth-generation and next-generation CAR-T cells. With the aid of transduced cytokines and the agonist of the T cell receptor, CAR-T cells self-activate through autocrine and paracrine mechanisms and stimulate host T cells to regain antitumor efficacy. In addition, blockade of the inhibitory receptor prevents the apoptosis induced by receptors such as PD-1, which reconstructs the host immune system. Furthermore, a BiTE facilitates CAR-T cell and host T cell infiltration into tumor sites and the interaction between T cells and tumor cells. For tumors with a strong TME, artificially transduced CXCRs guide CAR-T cells to the tumor and express chemokines at high levels, a key method by which the tumor modulates other immune cells and forms the TME. Last but not least, we illustrate switch receptors, which are assembled from the extracellular domain of the inhibitory receptor and the intracellular domain of the activating receptor

With the expansion of clinical trials, checkpoint inhibitor therapy has shown the ability to reverse the exhaustion of CAR-T cells in the short-term in relapsed patients and to shape the tumor environment through the inducible release of transgenic immune modifiers. CAR-T cell therapies based on these principles are known as fourth-generation CAR-T cell therapies [60]. In addition, with the exception of immune modifiers, T cell engagers and some membrane receptors are also transduced with second-generation CAR-T cells, which are generally called next-generation CAR-T cells. These CAR-T cells are not limited by T cell cytotoxicity and represent a powerful tool to re-establish the immune system after infusion. As the most highly anticipated advancement to date, fourth-generation and next-generation CAR-T cells CAR-T cells are divided into two categories, cells that utilize secreted molecules and cells that utilize membrane receptors (Fig. 2). Both types are described below.

#### Secreted molecules

The TME reduces the antitumor and proliferative activities of CAR-T cells. A sequence that encodes a secreted peptide is transfected into the T cells during manufacturing to improve the resistance of CAR-T cells to TME-induced effects and activate the immune system. When these CAR-T cells are activated, the sequence is transcribed and secretes an immune modifier into the extracellular fluid, where it not only persistently stimulates CAR-T cells to remain active and induce the formation of T memory cells but also reactivates the host immune system to respond to restimulation. In theory, the concentration of the secreted protein is related to the distribution of CAR-T cells and their activation levels; in other words, the secreted protein delivers drugs precisely to the tumor TME. Therefore, the current coding sequence includes a T cell engager, cytokines, and an agonist or inhibitor of cell receptors.

##### Bispecific T cell engager (BiTE) and bispecific antigens

BiTE and bispecific antigens have two different functions. One binds to the TAA, and the other has a high affinity for a T cell epitope, normally CD3, which is an adaptor that recruits T cells to the tumor. Compared to BiTE, the bispecific antigen contains a complete antigen structure through which Fc activates T cells, which may improve the CAR-T cell tumor-killing activity. In a clinical trial of AMG 420, the BCMA-CD3 BiTE used in CAR-T cell therapy for patients with R/R MM, the overall response rate was 31% (13/42), and 9 patients achieved CR, 2 achieved VGPR, and 2 achieved a partial response; the median response time was 8.4 months [61]. However, in the group of patients who received the maximum tolerated dose (400 μg/d), the response rate was 70% (*n* = 7 of 10). Five of these patients experienced an MRD-negative complete response, 1 achieved PR, and 1 achieved VGPR. All 7 patients responded during the first cycle, and some responses lasted > 1 year [62]. Combined with their “off the shelf” character, BiTEs are close to receiving clinical approval for use in patients with R/R MM. One possible explanation for the nonresponding cases may be the anergy of the T cells, although T cells were recruited to the TME. The response rate of the patients with R/R MM to CAR-T therapy is moderately high, with an 85% objective response rate, of which approximately 30% of the 33 patients exhibited a complete response [12]. In an attempt to salvage a patient who relapsed after anti-CD22 CAR-T cell therapy, blinatumomab successfully re-expanded the anti-CD22 CAR-T cells and induced complete remission, which prolonged the patient’s life span in this case [63]. Based on this outcome, the combination of BiTEs and CAR-T cell strengthens the effects of each treatment. The sequence encoding BiTE is transfected into T cells with the CAR sequence and BiTE-armored CAR-T cells are produced to take full advantage of CAR-T cell tumor infiltration and overcome the tumor heterogeneity. CD3/EGFR BiTEs were adapted for an anti-EGFRIII CAR-T cell treatment of neuroblastoma. The preclinical studies were promising in terms of both safety and efficacy and showed that EGFR, a tumor-associated antigen expressed on most epithelial cells, is able to be selected as a target of BiTE [64]. The potential explanation for this success is that EGFRIII is a tumor-specific antigen that only presents the direct activation signal to the CAR-T cells. This example represents the safe adaption of the widely expressed TAA, which provided CAR-T cells with a primary homing and recruitment signal to facilitate the infiltration of CAR-T and host T cells into tumors that had been activated by the CAR signal. For hematological malignancies, secreted BiTE-armored CAR-T cells have already shown efficacy against leukemia in mice [65]. BiTE or bispecific antigen-armored CAR-T cells may be another approach to overcome tumor heterogeneity and take full advantage of the TAAs that are expressed on normal cells as primary targets for T cells. Unfortunately, clinical trials of BiTE and CAR-T cell therapy have both indicated the possibility of severe side effects during treatment. In a clinical trial of blinatumomab as a treatment for B cell ALL and NHL, the pooled occurrence rate of grade ≥ 3 CRS was 0.04, and the pooled occurrence rate of grade ≥ 3 neurological events was 0.12 [66]. In another trial of anti-CD19 CAR-T cells for B cell ALL, NHL and CLL, 133 patients completed a toxicity assessment. CRS had developed in 71% of the patients (60% grades 1–2, 4% grade 3, and 8% grade ≥ 4). NT was observed in 40% of the patients (19% grades 1–2, 16% grade 3, and 5% grade ≥ 4) and grade ≥ 3. NT manifested at a median of 4.5 days after CRS onset. Therefore, the combination of BiTEs and CAR-T cells may require the mitigation of the possible side effects, a solution that must be obtained before BiTE-armored CAR-T cells can be adapted for use in the clinic. However, for solid hematological tumors, such as R/R lymphoma and R/R MM, the use of a secreted BiTE may be a strategy to enhance engraftment and promote infiltration to improve treatment efficiency.

##### Pro-inflammatory cytokines

Cytokines are the main regulators of inflammation in the normal immune state. However, the TME may attenuate the functions immune cells, such as defective APCs in the tumor milieu, tumor-associated macrophages (TAMs), and myeloid-derived suppressor cells (MDSCs), to inhibit them on contact, or it may secrete immunosuppressive molecules to prevent the immune system from efficiently responding to the tumor. T cell therapy is unable to achieve a favorable response in a solid tumor when the TME is actively working against it. Regarding hematological malignancies, in clinical trials of anti-CD19 CAR-T cell therapy for R/R B cell lymphoma, the concentration of IL-15 after CAR-T cell infusion was a strong predictor of the patients’ prognosis [67, 68]. Interleukin-7 (IL-7) and IL-15 enrich select early lineage cells and rescue T cell expansion; thus, IL-15 is a strong stimulator of the maintenance of T cell stemness [69]. Therefore, IL-7, IL-12, IL-15, and other proinflammatory cytokines have been combined with several immune therapies, including CAR-T cells, to endow CAR-T cells with a strong tumor-killing activity and the capacity to modulate the immune system. According to clinical trials, this strategy is effective at mitigating the effects of the TME, but the trials also exposed the disadvantages of these inflammatory mediators. For example, in primary clinical trials of IL-2 with anti-PSMA CAR-T cell therapy, CAR-T cell engraftment was greater than 20%, and IL-2 signaling was restrained. However, an increase in the dosage through I.V. administration might cause systematic toxicity, which may increase the risk of vascular hemorrhage. Therefore, the sequences encoding the selected molecules were added to the CAR sequence and transduced into the T cells. When these cytokine-armored CAR-T cells were activated, their stemness was retained, and they were able to resist the TME by activating autocrine and paracrine mechanisms, which transform a “cold” tumor into a “hot” one. Cytokine-armored CAR-T cells have also been shown to improve the effectiveness and persistence of the cells in treatments of some solid tumors and hematological malignancies [70–79]. Newly discovered molecules, such as IL-23, have also shown promise in improving CAR-T cell efficacy by increasing the persistence and anti-TME activity of CAR-T cells [80]. For cytokine-armored CAR-T cells, the concentration of the cytokine positively correlates with the number and activity level of the CAR-T cells, which may increase the accuracy and efficacy at the same time. In addition, these cytokines also promote the formation of T memory cells, which increase the persistence of CAR-T cells and reactivate the host T cells to respond to the tumors. However, for patients with hematological malignancies, the careful selection of the armored cytokine is required to prevent disease progression.

##### Agonists and blockers of T cell receptors

Intrinsic agonists of T cell receptors, such as 4-1BB and OX40, improve T cell proliferation and cytotoxicity, increasing the activity of T cells and their ability to induce an immune response to the tumor. In the intracellular domain, these agonists provide a second stimulatory signal for CAR-T cells. The efficiency of this strategy has been reported in preclinical studies, and the ligand of 4-1BB (4-1BBL) is currently one of the most commonly adopted costimulation domains. Compared with the fixed intracellular domain of the CAR, the secreted ligand confers resistance to the suppressive effect of the TME and reactivates T cells in a state of anergy to allow them to respond. Prior to the administration of CAR-T cell therapy, the ligand of the costimulation receptor is added to the vaccine to stimulate the T cells [81, 82]. For example, the efficiency and safety of a mAb against the ligand 4-1BB have been confirmed in clinical trials. The ratios of active T cells and memory T cells that were able to infiltrate the tumors were both increased in this clinical trial [83]. Moreover, the agonist of 4-1BB induces CD8+ T cells to upregulate enzyme-B and IFN-gamma during tumor killing and to maintain the stemness of Tem [43]. Based on these findings, therapies in which a specific ligand is transduced into CAR T cells during manufacturing, the so-called agonist-armored CAR T cells, are currently being investigated in trials for patients with R/R NHL and CLL. The initial results showed no significant difference in the efficiency or safety of the anti-CD19 4-1BBL-armored CAR-T cells. The overall CR rate was 57% (16/28), and the CR rates were higher in patients with large cell lymphoma (78%) than in patients with CLL (20%) [84]. The modest outcome for the patients with CLL might be related to the low dose administered. In the aforementioned clinical trial of anti-CD19 CAR-T cells as a treatment for CLL/SLL, an MRD-CR rate of 60% was obtained in 30 days at doses of 50 × 10e6 and 100 × 10e6; however, the anti-CD19 4-1BB-armored CAR-T cell trial administered only 5 × 10e6 CAR-T cells per patient. Therefore, the verification of the long-term response rate and its specific features will require more subjects and time. A phase I trial of CD19-targeted EGFRt/19-28z/4-1BBL-armored CAR-T cells in patients with relapsed or refractory CLL will begin soon [85]. Although CD137 (4-1BB) activates CAR-T cells and increases their proliferation and persistence, further exploration of the pathway that regulates metabolism and differentiation of T cells on these activating receptors will undoubtedly increase the accuracy and specificity of clinical applications for different types of hematological malignancies.

For most solid tumors, the main obstacle to effective treatment is the TME, which coerces the immune system to tolerate the tumor and provides an environment in which the tumor can grow. Among CAR-T cell therapies, a distinct population of T cells manifests anergy differently in patients who achieve a complete response and patients who are nonresponsive. The critical markers on T cells that determine their exhaustion are PD-1, LAG3, and TIM3 [3, 83]. The mAbs against PD1/PDL1 represent the most popular checkpoint inhibitor immune therapies and have been widely adopted in clinical trials for advanced tumors. For patients with hematological malignancies, classic HL exhibits an excellent response (ORR of approximately 87% and PFS of 86% at 24 weeks for classic HL) to the PD-1 blocker nivolumab [86], similar to MM and NHL, but not primary mediastinal B cell lymphoma (PMBCL), for which the response rate is somewhat modest [87]. The mAb against the PD-1 blocker is a consolidation drug used after ASCT for MM and HL that has also generated favorable data [88, 89]. Therefore, the inhibitor of PD-1 reverses the effects of the TME to some extent and helps prevent T cell exhaustion. Although disrupting the PD-1 receptor using the clustered regularly interspaced short palindromic repeats (CRISPR)/CAS9 system enables CAR-T cells to resist the TME and increases the tumor-killing efficiency [90], the secreted PD-1 blocker rescues the “bystander” T cells and reconstructs the immune system in patients [91–94]. The PD-1 blocker showed the ability to rescue PD-1-overexpressing (exhausted) CAR-T cells [95], but ongoing clinical trials will reveal whether the secreted PD-1 blocker is effective in CAR-T cells. Moreover, blockers of LAG3 and other inhibitory receptors are also being developed.

In contrast to cytokines, agonists and blockers of T cell receptors target only one specific receptor, decreasing the likelihood of uncontrollable inflammatory side effects; however, based on the experience of using inhibitors of the PD-1 pathway, the nonresponse rate is relatively high, even when patients show substantial disease progression. Therefore, the treatment still requires fundamental research, and more subjects are needed to explore the best modular structure.

### Membrane receptors

#### Chemokine receptors

Chemokines and their receptors, which are expressed on immune cells and noninnate immune cells such as epithelial cells, have important contributions to immune cell migration and infiltration. However, in the TME, IL-8 and the chemokine receptor CXCR2 exert deleterious effects on the recruitment and modulation of immune cells. For example, human hepatocellular carcinoma (HCC) tumor tissues and cell lines express the CXCR2 ligand at high levels to regulate TAMs. T cells intrinsically lack CXCR2. Therefore, the sequence that encodes CXCR2 is introduced during manufacturing to improve CAR-T cell homing to HCC tissues. This approach has generated favorable outcomes compared to cells without the trafficking chemokine receptor [96]. CXCR2 is also expressed on tumors, including melanoma, renal cell carcinoma, non-small-cell lung cancer, pancreatic tumors, breast tumors, and ovarian cancer [97]. Regarding hematological malignancies, although receptors such as CXCR4 are key molecules for MM, ALL, and CLL cell trafficking into and out of the bone marrow [97], more research is needed to determine whether they represent effective target chemokines and to ensure that they will not alter the niche for the bone marrow stem cells or misdirect CAR-T cells into attacking normal lymphoid organs. However, similar to BiTEs, the proper choice of the chemokine receptor might induce CAR-T cells to enter the tumor site, particularly solid tumors or tumors have assembled together, which increases the efficacy of the CAR-T cells.

#### Switch receptors

As explained above, CD28 is a promising costimulation domain, and PD-1 is one of the main checkpoint inhibitors in T cells. A preclinical study is combining the sequence of the ectodomain of PD-1, and the intracellular domain of CD28, which is called a “switch receptor.” When CAR-T cells are activated, these PD-1/CD28 receptors are expressed at high levels on the CAR-T cells, through which the TME was modified, and these cells were able to infiltrate tumors in vivo, particularly expressing PD-L1. A clinical trial was conducted with CD19 CAR-T cells expressing the PD-1/CD28 chimeric switch receptor in patients with R/R B cell lymphoma. The overall response rate was 58.8% (10/17), and the CR rate was 41.2% (7/17) [98]. Based on the same theory used in the clinical trial, a switch for inhibiting IL-4 and activating IL-7 or IL-21 has been tested in preclinical experiments and has also shown promising anti-TME activity [99]. Because the mechanism of the TME is more complex than we currently understand, the actual function of the switch requires further testing in different tumor models to avoid the overactivation of CAR-T cells and damage to the healthy tissue.

Currently, clinical trials on fourth-generation and next-generation CAR-T cell therapy are in progress worldwide, and the results of these studies are highly anticipated (Table 1). Clearly, fourth-generation and next-generation CAR-T cell therapy will provide hope to combat B cell malignancies and other hematological malignancies, even advanced solid tumors. However, most of the characteristics of fourth-generation and next-generation CAR-T cells used in the clinic remain unclear, and further endeavors are needed for the translation of fourth-generation and next-generation CAR-T cells into the clinic.

## Manufacturing technology

Most currently available CAR-T therapies are manufactured using lentiviruses. Due to the time-consuming manufacturing process, approximately 10% of patients die because their disease progresses before they can receive treatment. A decrease in the manufacturing time has therefore attracted considerable attention because it will enable patients with an aggressive disease to receive treatment and will help patients maintain the stem subtype ratio of T cells [100]. A new technology called FasT CAR-T decreases the manufacturing time to only 24 h and has been subjected to phase I clinical trials (NO: ChiCTR1900023212) at Xinqiao Hospital in China. Patients can be treated with this type of CAR-T cell within a short period, and the first trial resulted in all 10 patients achieving CR 4 weeks after the CAR-T cell infusion; 9 of these patients were also negative for MRD. The major adverse event was CRS, which was observed in 9 patients. Three patients experienced CAR-related encephalopathy syndrome (CRES) after CAR-T infusion. All the patients who developed CRS or CRES recovered after intervention (unpublished data). The latent risk of the viral infection and possible random insertions might lead to the loss or gain of function of T cells, which may lead to unlimited proliferation; a case report of improperly cultured leukemic CAR-B cells leading to fatal relapse has been published, which spurred research into nonvirus-mediated transduction methods [20]. Sleeping Beauty and PiggyBac transposition are nonvirus-based technologies that enable T cells to be processed in less time while producing a higher ratio of central memory T cells with limited DNA damage [101–104]. In theory, this process prolongs the persistence of CAR-T cells and avoids the latent risk of DNA damage. The initial outcomes of CAR-T cells manufactured using the Sleeping Beauty system based on a shorter culture time showed antitumor efficiency in 6 patients without eliciting clinically significant CRS [102]. PiggyBac has been used to manufacture HLA-matched anti-CD19 allo-CAR-T cells, and in a clinical trial, all 7 subjects achieved CR, suggesting similar safety and activity to CD19 CAR-T cells generated with viral vectors [105]. However, the long-term benefits must be verified through further clinical trials. In addition to decreasing the risks associated with virus vectors and the manufacturing time, manufacturing technology reformation is also regarded as an efficient method to lower the cost.

The evolution of manufacturing technology is not limited to CAR gene transduction. Gene editing technology has also been adopted to improve the ability of CAR-T cells to resist the TME and decrease the immunogenicity. The most popular pathway is the PD-1 pathway. The next-generation CAR-T cells include several gene editing methods such as CRISPR/CAS9 to disrupt the inhibitory effect of this pathway on CAR-T cells by silencing or knocking out the gene encoding PD-1. Based on the proven increase in the tumor-killing efficacy derived from the results of preclinical studies of several PD-1-disrupted CAR-T cell therapies for solid tumors [106, 107], the clinical trials of PD-1 knock out CAR-T cells have begun to enroll patients (Table 2). The primary outcome of a clinical trial of PD-1 KO engineered anti-MUC1 CART cells for the treatment of patients with advanced non-small-cell lung cancer (NSCLC) showed that 11 of the 20 assessed patients presented with stable disease, while 9 experienced disease progression. All patients experienced significant improvements in their symptoms after the infusion. However, the appropriate dosage and cycle remain to be explored [108]. Additionally, more specific editing was proposed to minimize the latent risk of off-target side-effects of gene editing. A reduction in the N-linked glycosylation of PD-1 may decrease PD-1 expression and relieve its inhibitory effects on CAR-T cells. Therefore, a preclinical study using an adenine base editor (ABE) to reduce PD-1 suppression by changing the glycosylated residue in CAR-T cells downregulated the expression of PD-1 in CAR-T cells and enhanced cytotoxic functions in vitro and in vivo [11]. Although silenced PD-1 promotes the efficacy of CAR-T cells, the negative effect on persistence may harm the total benefit of CAR-T cell therapy. In a preclinical study, better antitumor function was not presented by PD-1-blocked CAR-T cells in vitro or in vivo. PD-1 knock down might impair the antitumor potential of CAR-T cells because it inhibits T cell proliferation and accelerates the early differentiation of T cells and prevents effector T cells from differentiating into effector memory T cells. Thus, PD-1 might play an important role in maintaining the proper proliferation and differentiation of T cells, and PD-1 silencing would impair the antitumor function of T cells by inhibiting their proliferation [109]. Therefore, the next goal is to partially suppress the PD-1 signaling pathway to maintain the proliferation and differentiation of CAR-T cells while improving the efficacy and resistance to the TME. Additionally, with the exploration of the intracellular network, adjustments to the activation by altering the expression of transcription factors such as NR4A [110] may be more accurate, which also produced notable results in preclinical studies and revealed the future of the next-generation CAR-T cells based on epigenetic modifications. However, the risks associated with gene editing should always be a concern, as all products should remain vulnerable to chemotherapy or other therapies to ensure the control of the infused cells. A suicide gene should also be considered to ensure safety, as we do not completely understand the effects of gene editing.

**Table 2:** Clinical trials of fourth-generation/next-generation and gene-edited CAR-T cell therapies around the world

Number	Disease	Binding epitope	Armor/gene-edit	Location	Start time	Study completion	Number enrolled	Status
NCT04162119	MM	BCMA	PD-1Fc	Sanya, Hainan, China	2019/7/7	2022/10/10	30	Recruiting
NCT04109482	BPDCN,AML,HRMDS	CD123	Truncated EGFR	Durham, North Carolina, USA	2020/2/25	2026/4/1	126	Recruiting
NCT03778346	MM	CD138, integrin β7, CS1, CD38, and BCMA	IL7 and CCL19	Lishui, Zhejiang, China	2018/11/15	2022/12/31	30	Recruiting
NCT03929107	B cell lymphoma	CD19	IL-7 and CLL 19	Hangzhou, Zhejiang, China	2019/3/28	2022/4/30	80	Recruiting
NCT03258047	B cell lymphoma	CD19	PD1/CD28 switch-receptor	Hangzhou, Zhejiang, China	2017/9/15	2019/7/30	60	Active, not recruiting
NCT03208556	B cell lymphoma	CD19	PD1 shRNA-expressing cassette	Beijing, China	2017/6/21	2020/6/1	20	Recruiting
NCT03932955	B cell lymphoma	CD19	PD1/CD28 switch-receptor	Beijing, China	2019/5/1	2021/5/1	15	Recruiting
NCT04163302	B cell lymphoma	CD19	PD-1Fc	Sanya, Hainan, China	2019/7/7	2022/7/1	30	Recruiting
NCT03085173	B-CLL	CD19	4–1BBL	New York, USA	2017/3/15	2020/3/1	37	Recruiting
ChiCTR1900021295	DLBCL	CD19	Dominant negative PD1 molecule	Shanghai, China	2018/6/1	2022/6/1	5	Not mentioned
NCT03602157	HL CTCL	CD30	CCR4	Chapel Hill, North Carolina, USA	2018/12/12	2041/9/30	59	Recruiting
NCT03182816	Advanced solid tumor	EGFR	CTLA-4/PD-1 antibody	Ningbo, Zhejiang, China	2017/6/7	2019/4/20	40	Unknown
NCT02873390	Advanced solid tumor	EGFR	PD-1 antibody	Ningbo, Zhejiang, China	2016/8/1	2018/7/1	20	Unknown
NCT03635632	Neuroblastoma	GD2	IL-7 receptors	Houston, Texas, USA	2019/4/23	2037/12/1	64	Recruiting
NCT03721068	Neuroblastoma	GD2	IL-15	Chapel Hill, North Carolina, USA	2019/2/19	2039/6/19	18	Recruiting
NCT03030001	Advanced solid tumor	Mesothelin	PD-1 antibody	Ningbo, Zhejiang, China	2017/2/15	2019/2/1	40	Unknown
NCT03182803	Advanced solid tumor	Mesothelin	CTLA-4/PD-1 antibody	Ningbo, Zhejiang, China	2017/6/7	2019/4/20	40	Unknown
NCT03615313	Advanced solid tumor	Mesothelin	PD-1 antibodies	Shanghai, China	2018/8/6	2020/12/3	50	Recruiting
NCT03179007	Advanced solid tumor	MUC1	Anti-CTLA-4/PD-1	Ningbo, Zhejiang, China	2017/6/7	2019/4/20	40	Unknown
NCT02498912	Advanced solid tumor	MUC16	IL-12	New York, USA	2015/8/1	2020/8/1	30	Recruiting
NCT03932565	Advanced solid tumor	Nectin4	IL7 and CCL19, or IL12	Zhejiang, China	2019/2/13	2021/12/3	30	Recruiting
NCT04381741	DLBCL	CD19	IL7 and CCL19	Zhejiang, China	2020/9/1	2023/9/1	24	Recruiting
NCT03721068	Neuroblastoma	GD2	IL-15	North Carolina, USA	2019/2/19	2039/6/19	18	Recruiting
NCT04093648	Hepatoblastoma	Glypican 3	IL21 and IL15	United States	2020/1/1	2038/1/1	60	Not yet recruiting
NCT04185038	Central nervous system tumor	B7H3	Truncated EGFR	Washington, USA	2019/12/11	2041/5/1	70	Recruiting
NCT03208556	B cell lymphoma	CD19	PD1 shRNA-expressing cassette	Beijing, China	2016/7/1	2019/7/1	20	Recruiting
NCT03198546	Hepatocellular carcinoma	GPC3	Soluble TGFβ	Guangdong, China	2017/7/1	2022/8/1	30	Recruiting
NCT03706326	Advanced esophageal cancer	MUC1	PD-1 Knockout	Guangdong, China	2018/9/28	2021/9/28	20	Recruiting
NCT03525782	Non-small cell lung cancer	MUC1	PD-1 Knockout	Guangdong, China	2018/2/1	2022/1/31	60	Recruiting
NCT03545815	Solid tumor	Mesothelin	PD-1 Knockout	Beijing, China	2018/6/1	2020/6/30	10	Recruiting
NCT03298828	B-ALL and Buekitt lynphoma	CD19	PD-1 Knockout	Chongqing, china	2017/11/1	2022/10/1	30	Not yet recruiting

## The sources of T cells

Most of the currently available CAR-T cells are obtained from T cells harvested from patients, which are manufactured into CAR-T cells. However, patients with R/R hematological malignancies who are treated with CAR-T therapies usually have undergone a series of chemotherapies, some of whom suffered a frank relapse after HSCT. The high tumor burden and a deficiency in a population of T cells increase the difficulty of acquiring a sufficient number of qualified T cells upon apheresis, particularly for patients with T cell malignancies. For patients who have suitable donors, allo-CAR-T therapy has been shown to be suitable in phase I clinical trials and people receiving assistive allo-HSCT [111–114]. The speculation that allo-HSCT causes severe graft-versus-host disease (GVHD) was shown to be an unnecessary concern [115]. After a systematic review of 72 patients who received anti-CD19 allo-CAR-T cells after relapse of frank B cell malignancies, only 5 patients experienced GVHD [116, 117]. Furthermore, allo-CAR-T cells have the potential to facilitate the implantation for haploidentical HSCT (haplo-HSCT); for example, they successfully helped a 71-year-old patient in need of allo-transplantation [114]. After transplantation, anti-CD19 allo-CAR-T cells have also salvaged patients after frank B cell tumor relapse or prevented them from relapsing [112, 113]. As explained above, anti-CD123 CAR-T cells may damage the bone marrow stem cells while eliminating AML cells, rendering them of limited clinical use. However, a case study reported the use of donor-derived anti-CD123 CAR-T cells as part of a conditioning regimen for haplo-HSCT in a patient with FUS-ERG+ AML who relapsed within 3 months after the first allogeneic transplantation and failed to achieve CR multiple times or to manifest full donor chimerism [118]. However, allo-CAR-T cell therapy remains expensive and time-consuming, even for patients who meet the donor-matching criteria. For patients without matched donors, “off the shelf” CAR-T cells from healthy donors may be the only choice available. Primary universal CAR-T (U-CAR-T) cells derived from a healthy donor were eliminated too quickly to function during the first study examining their feasibility, in part because hostile host natural killer (NK) cells and T cells kill incompatible (including but not limited to the T cell receptor) infused cells. However, some examples are available in which U-CAR-T cells were successfully implanted into patients due to weak resistance from the patient’s immune system. In the ideal model, U-CAR-T cells survive when the target antigen on malignant cells provides sufficient stimulation. When malignant cells are eliminated and the host immune system recovers, U-CAR-T cells would then be eliminated. In addition to using a relatively strong preconditioning chemotherapy, several methods have been proposed to improve engraftment. The solution is based on the selection of specific subtypes of T cells that are able to avoid the host immune response. Among these subtypes, T cells that express innate EBV-specific T cell receptor (TCR) and γδ-T cell subtypes are promising and do not require gene editing. The mechanism by which donor T cells with an EBV-specific TCR escape host rejection is unknown but is apparently related to naive T cells. The clinical application of donor EBV-specific T cells indicates a low rate of GVHD in patients after HSCT. Anti-CD19 EBV-specific CAR-T cells have shown antitumor efficacy against CD19+ malignant B cells [119]. The primary results in terms of efficacy and safety of 19-28z CAR EBV-CTL showed CR in 70% (7/10) of the treated patients, including 100% (4/4) with NHL and 83% (5/6) of recipients with third-party cells, and CRS or neurotoxicity were not observed, providing hope to patients with no alternative treatments [120]. For γδ-T cells without the limitation of HLA matching, the most abundant γδ-T cells expressed the Vγ9 Vδ2 TCR that recognizes isopentenyl pyrophosphate (IPP), which is overproduced in cancer cells. Costimulation domain-CAR γδ-T cells have been considered a safer therapy because the CAR follows only one pathway to stimulate the immune response, and the cytotoxicity was limited to the reaction induced by the IPP antigen [121, 122]. Furthermore, a large expansion platform has been successfully established, which has the potential to make γδ-T cells an off-the-shelf product and a possible source of U-CAR-T cell therapy [123].

Another method to generate cells that avoid the host immune system is gene editing. Zinc finger nuclease (ZFN), transcription activator-like effector nuclease (TALEN), and CRISPR/CAS9 technologies are capable of knocking out the TCR and other rejection-related epitopes, such as CD52. Initial clinical trials (NCT 02746952 and NCT02808442) of anti-CD19 TALEN-edited TRAC and CD52-KO U-CAR-T cell therapy for adult and pediatric patients with B cell malignancies resulted in CR or CRi in 88% of the evaluable patients (14/16) by day 28 or day 42 after transplantation, and 86% (12/14) of these patients were MRD-negative, as determined by flow PCR or qPCR. Two patients failed to respond and showed no expansion of U-CAR-T cells [124]. Another type of allo-CAR-T cell called PBCAR0191 was manufactured by inserting an anti-CD19 CAR sequence into the TCR sequence to disrupt it, but it exhibited limited antitumor efficiency when administered to a low-dosage group [125]. Although U-CAR-T cells are derived from healthy donors, implantation, and secondary expansion are vital for the antitumor response. The U-CAR-T cell platform for T-ALL and MM treatment has shown efficacy in preclinical experiments [126, 127]. An ongoing trial of anti-CD7 U-CAR-T cells for patients with T cell lymphoma (NCT04264078) in Xinqiao Hospital indicated that 80% (4/5) of the patients displayed robust CAR-T cell expansion and achieved persistent MRD-CR. Genetically modified CAR-T cells can be used to avoid the main rejection from the host immune system and to increase engraftment, although the latent risk of the unlimited expansion of infused foreign CAR-T cells is a concern. However, U-CAR-T cells are derived from healthy donors, and thus, they are sensitive to glucocorticoids and chemotherapy. Furthermore, the modification is rather conservative, and host resistance is not completely eliminated; based on our experience, U-CAR T cells were undetectable in blood samples from recovered patients. In our opinion, current genetically modified U-CAR-T cell therapy is relatively safe. In summary, further studies are needed to explore the lymphocyte depletion protocol and appropriate dosage of U-CAR-T cell therapy to ensure antitumor efficacy. Furthermore, in contrast to auto-CAR-T cells, U-CAR-T cells are unlikely to persist in patients for a long time due to the heterogeneity involving more epitopes than we are able to delete. Therefore, treatments with U-CAR-T cells potentially represent a powerful method to achieve CR in time to benefit patients rather than serving as a persistent solution without further treatment. Potential follow-up treatments, which currently mainly include transplantation, require further exploration.

Additionally, immune cells, such as NK cells, with less immunogenicity and promising tumor-killing activity represent a potentially promising cell type to replace T cells and for use in universal immune cell therapy, and thus, researchers have focused on the development of CAR NK cells. However, the proliferation and persistence of NK cells are reduced compared with those of T cells. NK92 cells (a type of stable leukemic NK cells derived from patients with NK cell lymphoma) showed potential in eliminating several hematological and solid tumors in preclinical studies [128] , and the clinical grade manufacturing platform of NK92 cells has also been established to satisfy the required quality and quantity to overcome this problem [129]. A clinical trial of anti-CD33 CAR NK92 cells expressing the 4-1BB costimulation domain in the treatment of AML enrolled 3 patients, one of whom experienced MRD+ remission for a short period. The poor response rate may be attributed to the insufficient dosage and the limited number of subjects [130]. Moreover, the effective costimulation domain for NK cells is different from T cells. Therefore, the development of a stimulation domain as such 2B4 may be the solution to increase the engraftment and prolong persistence [131] . Additionally, the model used for the fourth generation might also be effective in CAR NK therapy. For example, the secreted IL-15 is also capable of increasing the response of patients to CAR NK cell therapy. With the enhancement, the aphesis of NK cells from healthy adult donors and induced by cord blood stem cells was an efficient and inexpensive source of CAR NK cell therapy [132, 133]. The CAR NK cells were successfully designed and tested in hematological malignancies and solid tumors in preclinical studies [134–136]. Clinical trials have been launched worldwide (Table 3). The primary outcome of anti-CD19 IL-15 secreted CAR NK cell therapy in patients with B cell lymphoma and CLL indicate that 8 (73%) of the 11 patients with relapsed or refractory CD19-positive cancers achieved a response; of these patients, 7 (4 with lymphoma and 3 with CLL) achieved CR, and 1 achieved remission of the Richter’s transformation component but had persistent CLL. Responses were rapid and observed within 30 days after the infusion at all dose levels. The infused CAR-NK cells expanded and persisted at low levels for at least 12 months [137]. In summary, CAR NK cell therapy showed promising potential as a universal cell therapy, and similar to UCAR-T cell therapy, the subsequent treatment should be considered.

**Table 3:** Clinical trials of CAR NK cell therapies around the world

Number	Disease	Binding epitope	Location	Start time	Study completion	Number enrolled	Status
NCT03692767	B cell lymphoma	CD19	NA	2019/3/1	2021/11/1	9	Not yet recruiting
NCT03690310	B cell lymphoma	CD22	NA	2019/3/1	2021/11/1	9	Not yet recruiting
NCT03824964	B cell lymphoma	CD19/CD22	NA	2019/2/1	2021/1/1	10	Not yet recruiting
NCT03056339	B lymphoid malignancies	CD19	Texas, USA	2017/6/21	2022/6/1	36	Recruiting
NCT03692663	Prostate cancer	PSMA	NA	2018/12/1	2021/12/1	9	Not yet recruiting
NCT03692637	Epithelial ovarian cancer	Mesothelin	NA	2019/3/1	2021/11/1	30	Not yet recruiting
NCT03383978	Glioblastoma	HER2	Frankfurt, Germany	2017/12/1	2020/8/1	30	Recruiting
NCT03940833	Multiple myeloma	BCMA	Jiangsu, China	2019/5/1	2022/5/1	20	Recruiting
NCT04004637	NK/T cell lymphoma, ALL	CD7	Henan, China	2019/6/1	2021/6/1	10	Recruiting
NCT03941457	Pancreatic cancer	ROBO1	Shanghai, China	2019/5/1	2022/5/1	9	Recruiting
NCT03940820	Solid tumor	ROBO1	Jiangsu, China	2019/5/1	2022/5/1	20	Recruiting
NCT03415100	Solid tumors	NKG2DL	Guangdong, China	2018/1/2	2019/12/1	30	Recruiting

## Conclusion and future perspectives

The fourth-generation and next-generation of CAR-T cells have been designed to increase their ability to kill tumors, extend their persistence in vivo, increase their ability to infiltrate solid tumor tissues, and increase their ability to modulate the immune microenvironment. The evolution of these strategies as treatments for hematological malignancies should include a consideration of the characteristics of different types of disease. For B cell ALL, the problem is not only based on methods to improve the initial efficacy but also involves needed improvements in CAR-T cell persistence and helping patients regain an immune response to the tumor. On the other hand, CAR-T cells for MM with solid tumors require increased trafficking and infiltration abilities and enhanced resistance to the TME. For AML, the specific target epitope is not easy to determine; therefore, all possible antigens, such as CD33 and CD123, may be necessary to combine transplantation and RNA-based transient CAR-T cells or costimulation CARs to salvage patients. Importantly, the interpretation of clinical trials may be limited by the enrollment of small numbers of subjects; therefore, ensuring the safety and efficacy of each variety of CAR-T cells requires clinical trials with a larger scope and long-term follow-up. In addition, a cure for hematological malignancies will never be achieved by relying on only one therapy; therefore, combined treatments and disease monitoring methods should be developed in parallel. Last but not least, the cost of CAR-T cell therapy should be reduced by technological advances and the development of universal CAR-T cell and CAR NK cell therapy to be accepted by the majority of patients and payors.

## Data Availability

Not applicable

## Abbreviations

ALL: Acute lymphoblastic leukemia
AML: Acute myeloid leukemia
BCMA: B cell mature antigen
BiTE: Bispecific T cell engager
CAR: Chimeric antigen receptor
CD: Cluster of differentiation
CLL: Chronic lymphoblastic leukemia
CR: Complete remission
CRES: CAR-related encephalopathy syndrome
CRISPR: Clustered regularly interspaced short palindromic repeats
CRS: Cytokine release syndrome
CXCR: CXC chemokine receptor
DLBCL: Diffuse large B cell lymphoma
EBV: Epstein-Barr virus
EGFR: Epidermal growth factor receptor
GVHD: Graft-versus-host disease
HCC: Hepatocellular carcinoma
HL: Hodgkin lymphoma
HSCT: Hematopoietic stem cell transplant
ITAM: Immunoreceptor tyrosine-based activation motif
mAb: Monoclonal antibody
MM: Multiple myeloma
NHL: Non-Hodgkin lymphoma
PMBCL: Primary mediastinal B cell lymphoma
PR: Partial remission
scFv: Single-chain variable fragment
TAA: Tumor-associated antigen
TALEN: Transcription activator-like effector nuclease
Tcm: Central memory T cell
TM: Transmembrane
TME: Tumor microenvironment
VGPR: Very good partial remission
ZFN: Zinc finger nuclease

## References

[1] Liu D (2019). CAR-T “the living drugs”, immune checkpoint inhibitors, and precision medicine: a new era of cancer therapy. J Hematol Oncol.

[2] Schuster SJ (2019). CD19-directed CAR T cells gain traction. Lancet Oncol.

[3] Zhang L-N, Song Y, Liu D (2018). CD19 CAR-T cell therapy for relapsed/refractory acute lymphoblastic leukemia: factors affecting toxicities and long-term efficacies. J Hematol Oncol.

[4] Locke FL, Ghobadi A, Jacobson CA, Miklos DB, Lekakis LJ, Oluwole OO (2019). Long-term safety and activity of axicabtagene ciloleucel in refractory large B-cell lymphoma (ZUMA-1): a single-arm, multicentre, phase 1–2 trial. Lancet Oncol..

[5] Schuster SJ, Bishop MR, Tam CS, Waller EK, Borchmann P, McGuirk JP (2019). Tisagenlecleucel in adult relapsed or refractory diffuse large B-cell lymphoma. N Engl J Med.

[6] Maude SL, Laetsch TW, Buechner J, Rives S, Boyer M, Bittencourt H (2018). Tisagenlecleucel in children and young adults with B-cell lymphoblastic leukemia. N Engl J Med.

[7] Siddiqi T, Soumerai JD, Dorritie KA, Stephens DM, Riedell PA, Arnason JE (2019). Rapid undetectable MRD (uMRD) responses in patients with relapsed/refractory (R/R) chronic lymphocytic leukemia/small lymphocytic lymphoma (CLL/SLL) treated with lisocabtagene maraleucel (liso-cel), a CD19-directed CAR T cell product: updated results from transcend CLL 004, a phase 1/2 study including patients with high-risk disease previously treated with ibrutinib. Blood.

[8] Zhao J, Lin Q, Song Y, Liu D (2018). Universal CARs, universal T cells, and universal CAR T cells. J Hematol Oncol.

[9] Liu D, Zhao J, Song Y (2019). Engineering switchable and programmable universal CARs for CAR T therapy. J Hematol Oncol.

[10] Zhao J, Song Y, Liu D (2019). Clinical trials of dual-target CAR T cells, donor-derived CAR T cells, and universal CAR T cells for acute lymphoid leukemia. J Hematol Oncol.

[11] Shi X, Zhang D, Li F, Zhang Z, Wang S, Xuan Y (2019). Targeting glycosylation of PD-1 to enhance CAR-T cell cytotoxicity. J Hematol Oncol.

[12] Raje N, Berdeja J, Lin Y, Siegel D, Jagannath S, Madduri D (2019). Anti-BCMA CAR T-cell therapy bb2121 in relapsed or refractory multiple myeloma. N Engl J Med.

[13] Rennert P, Su L, Dufort F, Birt A, Sanford T, Wu L (2019). A novel CD19-anti-CD20 bridging protein prevents and reverses CD19-negative relapse from CAR19 T cell treatment in vivo. Blood..

[14] Fry TJ, Shah NN, Orentas RJ, Stetler-Stevenson M, Yuan CM, Ramakrishna S (2018). CD22-targeted CAR T cells induce remission in B-ALL that is naive or resistant to CD19-targeted CAR immunotherapy. Nat Med.

[15] Wei J, Han X, Bo J, Han W (2019). Target selection for CAR-T therapy. J Hematol Oncol.

[16] Cummins KD, Frey N, Nelson AM, Schmidt A, Luger S, Isaacs RE (2017). Treating relapsed / refractory (RR) AML with biodegradable anti-CD123 CAR modified T cells. Blood..

[17] Tasian SK, Kenderian SS, Shen F, Li Y, Ruella M, Fix WC (2015). Efficient termination of CD123-redirected chimeric antigen receptor T cells for acute myeloid leukemia to mitigate toxicity. Blood..

[18] Kenderian SS, Ruella M, Shestova O, Klichinsky M, Aikawa V, Morrissette JJD (2015). CD33-specific chimeric antigen receptor T cells exhibit potent preclinical activity against human acute myeloid leukemia. Leukemia..

[19] Kim MY, Yu K-R, Kenderian SS, Ruella M, Chen S, Shin T-H (2018). Genetic inactivation of CD33 in hematopoietic stem cells to enable CAR T cell immunotherapy for acute myeloid leukemia. Cell.

[20] Ruella M, Xu J, Barrett DM, Fraietta JA, Reich TJ, Ambrose DE (2018). Induction of resistance to chimeric antigen receptor T cell therapy by transduction of a single leukemic B cell. Nat Med.

[21] Hossain N, Sahaf B, Abramian M, Spiegel JY, Kong K, Kim S (2018). Phase I experience with a bi-specific CAR targeting CD19 and CD22 in adults with B-cell malignancies. Blood..

[22] Shah NN, Zhu F, Taylor C, Schneider D, Krueger W, Worden A (2018). A phase 1 study with point-of-care manufacturing of dual targeted, tandem anti-CD19, anti-CD20 chimeric antigen receptor modified T (CAR-T) cells for relapsed, refractory, non-Hodgkin lymphoma. Blood..

[23] Amrolia PJ, Wynn R, Hough RE, Vora A, Bonney D, Veys P (2019). Phase I study of AUTO3, a bicistronic chimeric antigen receptor (CAR) T-cell therapy targeting CD19 and CD22, in pediatric patients with relapsed/refractory B-cell acute lymphoblastic leukemia (r/r B-ALL): Amelia study. Blood..

[24] Ardeshna KM, Marzolini MAV, Norman J, Al-Hajj M, Thomas S, Faulkner J (2019). Phase 1/2 study of AUTO3 the first bicistronic chimeric antigen receptor (CAR) targeting CD19 and CD22 followed by an anti-PD1 in patients with relapsed/refractory (r/r) diffuse large B cell lymphoma (DLBCL): results of cohort 1 and 2 of the Alexander study. Blood..

[25] Schultz LM, Muffly LS, Spiegel JY, Ramakrishna S, Hossain N, Baggott C (2019). Phase I trial using CD19/CD22 bispecific CAR T cells in pediatric and adult acute lymphoblastic leukemia (ALL). Blood..

[26] Dai H, Wu Z, Jia H, Tong C, Guo Y, Ti D (2020). Bispecific CAR-T cells targeting both CD19 and CD22 for therapy of adults with relapsed or refractory B cell acute lymphoblastic leukemia. J Hematol Oncol.

[27] Yang J, Li J, Zhang X, Lv F, Guo X, Wang Q (2018). A feasibility and safety study of CD19 and CD22 chimeric antigen receptors-modified T cell cocktail for therapy of B cell acute lymphoblastic leukemia. Blood..

[28] Yang J, Jiang P, Zhang X, Zhu X, Dong Q, He J (2019). Anti-CD19/CD22 dual CAR-T therapy for refractory and relapsed B-cell acute lymphoblastic leukemia. Blood..

[29] Gardner R, Annesley C, Finney O, Summers C, Lamble AJ, Rivers J (2018). Early clinical experience of CD19 x CD22 dual specific CAR T cells for enhanced anti-leukemic targeting of acute lymphoblastic leukemia. Blood..

[30] Wang N, Hu X, Cao W, Li C, Xiao Y, Cao Y (2020). Efficacy and safety of CAR19/22 T-cell cocktail therapy in patients with refractory/relapsed B-cell malignancies. Blood..

[31] Yan Z, Cao J, Cheng H, Qiao J, Zhang H, Wang Y (2019). A combination of humanised anti-CD19 and anti-BCMA CAR T cells in patients with relapsed or refractory multiple myeloma: a single-arm, phase 2 trial. Lancet Haematol.

[32] Li C, Mei H, Hu Y, Guo T, Liu L, Jiang H (2019). A bispecific CAR-T cell therapy targeting BCMA and CD38 for relapsed/refractory multiple myeloma: updated results from a phase 1 dose-climbing trial. Blood..

[33] Ritchie DS, Neeson PJ, Khot A, Peinert S, Tai T, Tainton K (2013). Persistence and efficacy of second generation CAR T cell against the LeY antigen in acute myeloid leukemia. Mol Ther.

[34] Wang J, Mou N, Yang Z, Li Q, Jiang Y, Meng J, et al. Efficacy and safety of humanized anti-CD19-CAR-T therapy following intensive lymphodepleting chemotherapy for refractory/relapsed B acute lymphoblastic leukaemia. Br J Haematol. n/a. Available from: https://onlinelibrary.wiley.com/doi/abs/10.1111/bjh.16623. [cited 2020 Apr 15]. 10.1111/bjh.16623 PMC7687133 32232846

[35] Hucks GE, Barrett D, Rheingold SR, Aplenc R, Teachey DT, Callahan C (2017). Humanized chimeric antigen receptor (CAR)-modified T cells targeting CD19 induce remissions in children and young adults with relapsed/refractory lymphoblastic leukemia/lymphoma. Cytotherapy..

[36] Yang F, Zhang J, Zhang X, Tian M, Wang J, Kang L (2019). Delayed remission following sequential infusion of humanized CD19- and CD22-modified CAR-T cells in a patient with relapsed/refractory acute lymphoblastic leukemia and prior exposure to murine-derived CD19-directed CAR-T cells. Onco Targets Ther.

[37] Lohmueller JJ, Ham JD, Kvorjak M, Finn OJ (2018). mSA2 affinity-enhanced biotin-binding CAR T cells for universal tumor targeting. OncoImmunology..

[38] Cho JH, Collins JJ, Wong WW (2018). Universal chimeric antigen receptors for multiplexed and logical control of T cell responses. Cell.

[39] Watanabe N, Bajgain P, Sukumaran S, Ansari S, Heslop HE, Rooney CM (2016). Fine-tuning the CAR spacer improves T-cell potency. OncoImmunology..

[40] Bishop DC, Xu N, Tse B, O’Brien TA, Gottlieb DJ, Dolnikov A (2018). PiggyBac-engineered T cells expressing CD19-specific CARs that lack IgG1 fc spacers have potent activity against B-ALL xenografts. Mol Ther.

[41] Alabanza L, Pegues M, Geldres C, Shi V, Wiltzius JJW, Sievers SA (2017). Function of novel anti-CD19 chimeric antigen receptors with human variable regions is affected by hinge and transmembrane domains. Mol Ther.

[42] Casucci M, Falcone L, Camisa B, Norelli M, Porcellini S, Stornaiuolo A (2018). Extracellular NGFR spacers allow efficient tracking and enrichment of fully functional CAR-T cells co-expressing a suicide gene. Front Immunol.

[43] Guedan S, Posey AD, Shaw C, Wing A, Da T, Patel PR (2018). Enhancing CAR T cell persistence through ICOS and 4-1BB costimulation. JCI Insight.

[44] Schneider D, Xiong Y, Wu D, Dropulic B, Orentas R (2017). Abstract 3746: plasma membrane spanning and linker-domains from tumor necrosis factor receptor superfamily (TNFRSF) proteins provide novel functionality to chimeric antigen receptors (CARs) expressed in human T cells. Cancer Res.

[45] Caimi PF, Reese J, Otegbeye F, Schneider D, Chamoun K, Boughan KM (2019). Phase 1 trial of anti-CD19 chimeric antigen receptor T (CAR-T) cells with tumor necrosis alfa receptor superfamily 19 (TNFRSF19) transmembrane domain. JCO..

[46] Caimi P, Reese JS, Otegbeye F, Schneider D, Bakalarz KL, Boughan KM (2020). On site manufacture of antiCD19 CAR-T cells. Responses in subjects with rapidly progressive refractory lymphomas. Biol Blood Marrow Transplant.

[47] Ying Z, Huang XF, Xiang X, Liu Y, Kang X, Song Y (2019). A safe and potent anti-CD19 CAR T cell therapy. Nat Med.

[48] Guedan S, Chen X, Madar A, Carpenito C, McGettigan SE, Frigault MJ (2014). ICOS-based chimeric antigen receptors program bipolar TH17/TH1 cells. Blood..

[49] Song D-G, Ye Q, Poussin M, Harms GM, Figini M, Powell DJ (2012). CD27 costimulation augments the survival and antitumor activity of redirected human T cells in vivo. Blood..

[50] Lu P, Lu X, Zhang X, Xiong M, Zhang J, Zhou X (2018). Which is better in CD19 CAR-T treatment of r/r B-ALL, CD28 or 4-1BB? A parallel trial under the same manufacturing process. JCO.

[51] Kawalekar OU, O’Connor RS, Fraietta JA, Guo L, McGettigan SE, Posey AD (2016). Distinct signaling of coreceptors regulates specific metabolism pathways and impacts memory development in CAR T cells. Immunity..

[52] Long AH, Haso WM, Shern JF, Wanhainen KM, Murgai M, Ingaramo M (2015). 4-1BB costimulation ameliorates T cell exhaustion induced by tonic signaling of chimeric antigen receptors. Nat Med.

[53] Quintarelli C, Orlando D, Boffa I, Guercio M, Polito VA, Petretto A, et al. Choice of costimulatory domains and of cytokines determines CAR T-cell activity in neuroblastoma. Oncoimmunology. 2018;7 Available from: https://www.ncbi.nlm.nih.gov/pmc/articles/PMC5980417/. [cited 2020 Feb 23]. 10.1080/2162402X.2018.1433518 PMC5980417 29872565

[54] Gomes-Silva D, Mukherjee M, Srinivasan M, Krenciute G, Dakhova O, Zheng Y (2017). Tonic 4-1BB costimulation in chimeric antigen receptors impedes T cell survival and is vector-dependent. Cell Rep.

[55] Gomes da Silva D, Mukherjee M, Srinivasan M, Dakhova O, Liu H, Grilley B (2016). Direct comparison of in vivo fate of second and third-generation CD19-specific chimeric antigen receptor (CAR)-T cells in patients with B-cell lymphoma: reversal of toxicity from tonic signaling. Blood.

[56] Tang X-Y, Sun Y, Zhang A, Hu G-L, Cao W, Wang D-H (2016). Third-generation CD28/4-1BB chimeric antigen receptor T cells for chemotherapy relapsed or refractory acute lymphoblastic leukaemia: a non-randomised, open-label phase I trial protocol. BMJ Open.

[57] Enblad G, Karlsson H, Gammelgård G, Wenthe J, Lövgren T, Amini RM (2018). A phase I/IIa trial using CD19-targeted third-generation CAR T cells for lymphoma and leukemia. Clin Cancer Res.

[58] Weng J, Lai P, Qin L, Lai Y, Jiang Z, Luo C (2018). A novel generation 1928zT2 CAR T cells induce remission in extramedullary relapse of acute lymphoblastic leukemia. J Hematol Oncol.

[59] Konstorum A, Vella AT, Adler AJ, Laubenbacher RC (2019). A mathematical model of combined CD8 T cell costimulation by 4-1BB (CD137) and OX40 (CD134) receptors. Sci Rep.

[60] Chmielewski M, Abken H (2015). TRUCKs: the fourth generation of CARs. Expert Opin Biol Ther.

[61] Topp MS, Duell J, Zugmaier G, Attal M, Moreau P, Langer C (2019). Evaluation of AMG 420, an anti-BCMA bispecific T-cell engager (BiTE) immunotherapy, in R/R multiple myeloma (MM) patients: updated results of a first-in-human (FIH) phase I dose escalation study. JCO..

[62] Topp MS, Duell J, Zugmaier G, Attal M, Moreau P, Langer C (2020). Anti–B-cell maturation antigen BiTE molecule AMG 420 induces responses in multiple myeloma. J Clin Oncol.

[63] Shalabi H, Koegel A, Ponduri A, Qin H, Salem D, Stetler-Stevenson M, et al. Case report: impact of BITE on CAR -T cell expansion. Adv Cell Gene Ther. 2019;2 Available from: https://onlinelibrary.wiley.com/doi/abs/10.1002/acg2.50. [cited 2020 Feb 23].

[64] Choi BD, Yu X, Castano AP, Bouffard AA, Schmidts A, Larson RC (2019). CAR-T cells secreting BiTEs circumvent antigen escape without detectable toxicity. Nat Biotechnol.

[65] Liu X, Barrett DM, Jiang S, Fang C, Kalos M, Grupp SA (2016). Improved anti-leukemia activities of adoptively transferred T cells expressing bispecific T-cell engager in mice. Blood Cancer J.

[66] Yu J, Wang W, Huang H (2019). Efficacy and safety of bispecific T-cell engager (BiTE) antibody blinatumomab for the treatment of relapsed/refractory acute lymphoblastic leukemia and non-Hodgkin’s lymphoma: a systemic review and meta-analysis. Hematology..

[67] Kochenderfer JN, Somerville RPT, Lu T, Shi V, Bot A, Rossi J (2017). Lymphoma remissions caused by anti-CD19 chimeric antigen receptor T cells are associated with high serum interleukin-15 levels. J Clin Oncol.

[68] Fraietta JA, Lacey SF, Orlando EJ, Pruteanu-Malinici I, Gohil M, Lundh S (2018). Determinants of response and resistance to CD19 chimeric antigen receptor (CAR) T cell therapy of chronic lymphocytic leukemia. Nat Med.

[69] Singh N, Perazzelli J, Grupp SA, Barrett DM (2016). Early memory phenotypes drive T cell proliferation in patients with pediatric malignancies. Sci Transl Med.

[70] Chen Y, Sun C, Landoni E, Metelitsa L, Dotti G, Savoldo B (2019). Eradication of neuroblastoma by T cells redirected with an optimized GD2-specific chimeric antigen receptor and interleukin-15. Clin Cancer Res.

[71] Yeku OO, Purdon TJ, Koneru M, Spriggs D, Brentjens RJ (2017). Armored CAR T cells enhance antitumor efficacy and overcome the tumor microenvironment. Sci Rep.

[72] Chou C, Fraessle S, Steinmetz R, Hawkins RM, Phi T-D, Busch D (2019). Combination of NKTR-255, a polymer conjugated human IL-15, with CD19 CAR T cell immunotherapy in a preclinical lymphoma model. Blood..

[73] Ataca Atilla P, Tashiro H, McKenna MK, Srinivasan M, Simons BW, Stevens AM (2019). Enhancing the effect of CLL-1 CAR T cells with interleukin-15 for treatment of acute myeloid leukemia. Blood..

[74] Perna SK, Pagliara D, Mahendravada A, Liu H, Brenner MK, Savoldo B (2014). Interleukin-7 mediates selective expansion of tumor-redirected cytotoxic T lymphocytes (CTLs) without enhancement of regulatory T-cell inhibition. Clin Cancer Res.

[75] Koneru M, Purdon TJ, Spriggs D, Koneru S, Brentjens RJ (2015). IL-12 secreting tumor-targeted chimeric antigen receptor T cells eradicate ovarian tumors *in vivo*. OncoImmunology..

[76] Chinnasamy D, Yu Z, Kerkar SP, Zhang L, Morgan RA, Restifo NP (2012). Local delivery of lnterleukin-12 using T cells targeting VEGF receptor-2 eradicates multiple vascularized tumors in mice. Clin Cancer Res.

[77] Pegram HJ, Lee JC, Hayman EG, Imperato GH, Tedder TF, Sadelain M (2012). Tumor-targeted T cells modified to secrete IL-12 eradicate systemic tumors without need for prior conditioning. Blood..

[78] Koneru M, O’Cearbhaill R, Pendharkar S, Spriggs DR, Brentjens RJ (2015). A phase I clinical trial of adoptive T cell therapy using IL-12 secreting MUC-16ecto directed chimeric antigen receptors for recurrent ovarian cancer. J Transl Med.

[79] You F, Jiang L, Zhang B, Lu Q, Zhou Q, Liao X (2016). Phase 1 clinical trial demonstrated that MUC1 positive metastatic seminal vesicle cancer can be effectively eradicated by modified anti-MUC1 chimeric antigen receptor transduced T cells. Sci China Life Sci.

[80] Ma X, Shou P, Smith C, Chen Y, Du H, Sun C, et al. Interleukin-23 engineering improves CAR T cell function in solid tumors. Nat Biotechnol. 2020:1–12. 10.1038/s41587-019-0398-2 PMC7466194 32015548

[81] Ragonnaud E, Andersson A-MC, Pedersen AE, Laursen H, Holst PJ (2016). An adenoviral cancer vaccine co-encoding a tumor associated antigen together with secreted 4-1BBL leads to delayed tumor progression. Vaccine..

[82] Fromm G, de Silva S, Giffin L, Xu X, Rose J, Schreiber TH (2016). Gp96-Ig/Costimulator (OX40L, ICOSL, or 4-1BBL) combination vaccine improves T-cell priming and enhances immunity, memory, and tumor elimination. Cancer Immunol Res.

[83] Chester C, Sanmamed MF, Wang J, Melero I (2018). Immunotherapy targeting 4-1BB: mechanistic rationale, clinical results, and future strategies. Blood..

[84] Palomba ML, Batlevi C, Riviere I, Senechal B, Wang X, Yang J (2019). A phase I first-in-human clinical trial of CD19-targeted 19-28Z/4-1BBL “armored” CAR T cells in patients with relapsed or refractory NHL and CLL including richter transformation: S1634. HemaSphere..

[85] Park JH, Riviere I, Wang X, Senechal B, Bernal Y, Halton E (2017). A phase I trial of CD19-targeted EGFRt/19-28z/4-1BBL armored chimeric antigen receptor (CAR) modified T cells in patients with relapsed or refractory chronic lymphocytic leukemia. J Clin Oncol.

[86] Ansell SM, Lesokhin AM, Borrello I, Halwani A, Scott EC, Gutierrez M (2015). PD-1 blockade with nivolumab in relapsed or refractory Hodgkin’s lymphoma. N Engl J Med.

[87] Lesokhin AM, Ansell SM, Armand P, Scott EC, Halwani A, Gutierrez M (2016). Nivolumab in patients with relapsed or refractory hematologic malignancy: preliminary results of a phase Ib study. JCO..

[88] Armand P, Chen Y-B, Redd RA, Joyce RM, Bsat J, Jeter E (2019). PD-1 blockade with pembrolizumab for classical Hodgkin lymphoma after autologous stem cell transplantation. Blood..

[89] Biran N, Andrews T, Feinman R, Vesole DH, Richter JR, Zenreich J (2017). A phase II trial of the anti -PD-1 monoclonal antibody pembrolizumab (MK-3475) + lenalidomide + dexamethasone as post autologous stem cell transplant consolidation in patients with high-risk multiple myeloma. Blood..

[90] Rupp LJ, Schumann K, Roybal KT, Gate RE, Ye CJ, Lim WA (2017). CRISPR/Cas9-mediated PD-1 disruption enhances anti-tumor efficacy of human chimeric antigen receptor T cells. Sci Rep.

[91] Linot C, Saini J, Adusumilli PS (2020). Sustained, cell-intrinsic versus intermittent, cell-extrinsic checkpoint blockade in solid tumor CAR T-cell therapy. JCO..

[92] Chen N, Morello A, Tano Z, Adusumilli PS (2017). CAR T-cell intrinsic PD-1 checkpoint blockade: a two-in-one approach for solid tumor immunotherapy. OncoImmunology..

[93] Rafiq S, Yeku OO, Jackson HJ, Purdon TJ, van Leeuwen DG, Drakes DJ (2018). Targeted delivery of a PD-1-blocking scFv by CAR-T cells enhances anti-tumor efficacy in vivo. Nat Biotechnol.

[94] Nakajima M, Sakoda Y, Adachi K, Nagano H, Tamada K (2019). Improved survival of chimeric antigen receptor-engineered T ( CAR -T) and tumor-specific T cells caused by anti-programmed cell death protein 1 single-chain variable fragment-producing CAR -T cells. Cancer Sci.

[95] Zhang R, Deng Q, Jiang Y-Y, Zhu H-B, Wang J, Zhao M-F (2019). Effect and changes in PD-1 expression of CD19 CAR-T cells from T cells highly expressing PD-1 combined with reduced-dose PD-1 inhibitor. Oncol Rep.

[96] Liu G, Rui W, Zheng H, Huang D, Yu F, Zhang Y, et al. CXCR2-modified CAR-T cells have enhanced trafficking ability that improves treatment of hepatocellular carcinoma. Eur J Immunol. 2020:eji.201948457. 10.1002/eji.201948457 31981231

[97] Perera LP, Zhang M, Nakagawa M, Petrus MN, Maeda M, Kadin ME (2017). Chimeric antigen receptor modified T cells that target chemokine receptor CCR4 as a therapeutic modality for T-cell malignancies: PERERA et al. Am J Hematol.

[98] Liu H, Lei W, Zhang C, Yang C, Wei J, Guo Q (2019). A phase I trial using CD19 CAR-T expressing PD-1/CD28 chimeric switch-receptor for refractory or relapsed B-cell lymphoma. JCO..

[99] Wang Y, Jiang H, Luo H, Sun Y, Shi B, Sun R (2019). An IL-4/21 inverted cytokine receptor improving CAR-T cell potency in immunosuppressive solid-tumor microenvironment. Front Immunol.

[100] Caruso HG, Tanaka R, Liang J, Ling X, Sabbagh A, Henry VK (2019). Shortened ex vivo manufacturing time of EGFRvIII-specific chimeric antigen receptor (CAR) T cells reduces immune exhaustion and enhances antiglioma therapeutic function. J Neuro-Oncol.

[101] Barnett BE, Hermanson DL, Smith JB, Wang X, Tan Y, Martin CE (2016). piggyBac™-produced CAR-T cells exhibit stem-cell memory phenotype. Target.

[102] Kebriaei P, Huls H, Neel SL, Olivares S, Orozco AF, Su S (2017). Shortening the time to manufacture CAR+ T cells with sleeping beauty system supports T-cell engraftment and anti-tumor effects in patients with refractory CD19+ tumors. Blood..

[103] Clauss J, Obenaus M, Miskey C, Ivics Z, Izsvák Z, Uckert W (2018). Efficient non-viral T-cell engineering by *Sleeping Beauty* minicircles diminishing DNA toxicity and miRNAs silencing the endogenous T-cell receptors. Hum Gene Ther.

[104] Morita D, Nishio N, Saito S, Tanaka M, Kawashima N, Okuno Y (2018). Enhanced expression of anti-CD19 chimeric antigen receptor in piggyBac transposon-engineered T cells. Mol Ther Methods Clin Dev.

[105] Bishop DC, Clancy LE, Burgess J, Mathew G, Atkins E, Advic S (2019). Matched sibling donor-derived piggybac CAR19 T cells induce remission of relapsed/refractory CD19+ malignancy following haematopoietic stem cell transplant. Cytotherapy..

[106] Zhu H, You Y, Shen Z, Shi L. EGFRvIII-CAR-T cells with PD-1 knockout have improved anti-glioma activity. Pathol Oncol Res. 2020; Available from: 10.1007/s12253-019-00759-1. [Cited 2020 May 14]. 10.1007/s12253-019-00759-1 31989402

[107] Hu W, Zi Z, Jin Y, Li G, Shao K, Cai Q (2019). CRISPR/Cas9-mediated PD-1 disruption enhances human mesothelin-targeted CAR T cell effector functions. Cancer Immunol Immunother.

[108] Lin Y, Chen S, Zhong S, An H, Yin H, McGowan E (2019). 35O - phase I clinical trial of PD-1 knockout anti-MUC1 CAR-T cells in the treatment of patients with non-small cell lung cancer. Ann Oncol.

[109] Wei J, Luo C, Wang Y, Guo Y, Dai H, Tong C (2019). PD-1 silencing impairs the anti-tumor function of chimeric antigen receptor modified T cells by inhibiting proliferation activity. J ImmunoTherapy Cancer.

[110] Chen J, López-Moyado IF, Seo H, Lio C-WJ, Hempleman LJ, Sekiya T (2019). NR4A transcription factors limit CAR T cell function in solid tumours. Nature..

[111] Wen S, Niu Z, Xing L, Wang Y, Li H, Kuang N (2018). CAR-T bridging to Allo-HSCT as a treatment strategy for relapsed adult acute B-lymphoblastic leukemia: a case report. BMC Cancer.

[112] Liu J, Zhong JF, Zhang X, Zhang C (2017). Allogeneic CD19-CAR-T cell infusion after allogeneic hematopoietic stem cell transplantation in B cell malignancies. J Hematol Oncol.

[113] Chen Y, Cheng Y, Suo P, Yan C, Wang Y, Chen Y (2017). Donor-derived CD19-targeted T cell infusion induces minimal residual disease-negative remission in relapsed B-cell acute lymphoblastic leukaemia with no response to donor lymphocyte infusions after haploidentical haematopoietic stem cell transplantation. Br J Haematol.

[114] Cai B, Guo M, Wang Y, Zhang Y, Yang J, Guo Y (2016). Co-infusion of haplo-identical CD19-chimeric antigen receptor T cells and stem cells achieved full donor engraftment in refractory acute lymphoblastic leukemia. J Hematol Oncol.

[115] Jacoby E, Yang Y, Qin H, Chien CD, Kochenderfer JN, Fry TJ (2016). Murine allogeneic CD19 CAR T cells harbor potent antileukemic activity but have the potential to mediate lethal GVHD. Blood..

[116] Anwer F, Shaukat A-A, Zahid U, Husnain M, McBride A, Persky D (2017). Donor origin CAR T cells: graft versus malignancy effect without GVHD, a systematic review. Immunotherapy..

[117] Ghosh A, Smith M, James SE, Davila ML, Velardi E, Argyropoulos KV (2017). Donor CD19 CAR T cells exert potent graft-versus-lymphoma activity with diminished graft-versus-host activity. Nat Med.

[118] Yao S, Jianlin C, Yarong L, Botao L, Qinghan W, Hongliang F (2019). Donor-derived CD123-targeted CAR T cell serves as a RIC regimen for haploidentical transplantation in a patient with FUS-ERG+ AML. Front Oncol.

[119] Shen RR, Pham CD, Wu M, Munson DJ, Aftab BT (2019). CD19 chimeric antigen receptor (CAR) engineered epstein-barr virus (EBV) specific T cells – an off-the-shelf, allogeneic CAR T-cell immunotherapy platform. Cytotherapy..

[120] Curran KJ, Sauter CS, Kernan NA, Prockop SE, Boulad F, Perales M (2020). Durable remission following “off-the-shelf” chimeric antigen receptor (CAR) T-cells in patients with relapse/refractory (R/R) B-cell malignancies. Biol Blood Marrow Transplant.

[121] Fisher J, Abramowski P, Wisidagamage Don ND, Flutter B, Capsomidis A, Cheung GW-K (2017). Avoidance of on-target off-tumor activation using a co-stimulation-only chimeric antigen receptor. Mol Ther.

[122] Parihar R (2017). Sensing bad: are co-stimulatory CAR-expressing γδ T cells safer?. Mol Ther.

[123] Xiao L, Chen C, Li Z, Zhu S, Tay JC, Zhang X (2018). Large-scale expansion of Vγ9Vδ2 T cells with engineered K562 feeder cells in G-rex vessels and their use as chimeric antigen receptor–modified effector cells. Cytotherapy..

[124] Benjamin R, Graham C, Yallop D, Jozwik A, Ciocarlie O, Jain N (2018). Preliminary data on safety, cellular kinetics and anti-leukemic activity of UCART19, an allogeneic anti-CD19 CAR T-cell product, in a pool of adult and pediatric patients with high-risk CD19+ relapsed/refractory B-cell acute lymphoblastic leukemia. Blood..

[125] Jacobson CA, Herrera AF, Budde LE, DeAngelo DJ, Heery C, Stein A (2019). Initial findings of the phase 1 trial of PBCAR0191, a CD19 targeted allogeneic CAR-T cell therapy. Blood..

[126] Cranert SA, Richter M, Tong M, Weiss L, Tan Y, Ostertag EM (2019). Manufacture of an allogeneic CAR-T stem cell memory product candidate for multiple myeloma, P-Bcma-ALLO1, is robust, reproducible and highly scalable. Blood..

[127] Gehrke JM, Edwards A, Murray RC, Shaw A, Poh Y-C, Smith S (2019). Highly efficient multiplexed base editing with minimized off-targets for the development of universal CAR-T cells to treat pediatric T-ALL. Blood..

[128] Ao X, Yang Y, Li W, Tan Y, Guo W, Ao L (2019). Anti-αFR CAR-engineered NK-92 cells display potent cytotoxicity against αFR-positive ovarian cancer. J Immunother.

[129] Nowakowska P, Romanski A, Miller N, Odendahl M, Bonig H, Zhang C, Seifried E, Wels WS, Tonn T (2018). Clinical grade manufacturing of genetically modified, CAR-expressing NK-92 cells for the treatment of ErbB2-positive malignancies. Cancer Immunol Immunother.

[130] Tang X, Yang L, Li Z, Nalin AP, Dai H, Xu T (2018). First-in-man clinical trial of CAR NK-92 cells: safety test of CD33-CAR NK-92 cells in patients with relapsed and refractory acute myeloid leukemia. Am J Cancer Res.

[131] Xu Y, Liu Q, Zhong M, Wang Z, Chen Z, Yu Z, Xing H, Zheng T, Tang K, Liao X, Rao Q, Wang M, Wang J. 2B4 costimulatory domain enhancing cytotoxic ability of anti-CD5 chimeric antigen receptor engineered natural killer cells against T cell malignancies. J Hematol Oncol. 2019;12(1). 10.1186/s13045-019-0732-7 PMC6524286 31097020

[132] Quintarelli C, Sivori S, Caruso S, Carlomagno S, Falco M, Boffa I, Orlando D, Guercio M, Abbaszadeh Z, Sinibaldi M, Di Cecca S, Camera A, Cembrola B, Pitisci A, Andreani M, Vinti L, Gattari S, Del Bufalo F, Algeri M, Li Pira G, Moseley A, De Angelis B, Moretta L, Locatelli F (2020). Efficacy of third-party chimeric antigen receptor modified peripheral blood natural killer cells for adoptive cell therapy of B-cell precursor acute lymphoblastic leukemia. Leukemia.

[133] Liu E, Tong Y, Dotti G, Shaim H, Savoldo B, Mukherjee M, Orange J, Wan X, Lu X, Reynolds A, Gagea M, Banerjee P, Cai R, Bdaiwi MH, Basar R, Muftuoglu M, Li L, Marin D, Wierda W, Keating M, Champlin R, Shpall E, Rezvani K (2018). Cord blood NK cells engineered to express IL-15 and a CD19-targeted CAR show long-term persistence and potent antitumor activity. Leukemia.

[134] Leivas A, Rio P, Mateos R, Paciello ML, Garcia-Ortiz A, Fernandez L, Perez-Martinez A, Lee DA, Powell DJ, Valeri A, Martinez-Lopez J (2018). NKG2D-CAR transduced primary natural killer cells efficiently target multiple myeloma cells. Blood.

[135] Reighard SD, Cranert SA, Rangel KM, Ali A, Gyurova IE, de la Cruz-Lynch AT, Tuazon JA, Khodoun MV, Kottyan LC, Smith DF, Brunner HI, Waggoner SN (2020). Therapeutic targeting of follicular T cells with chimeric antigen receptor-expressing natural killer cells. Cell Rep Med.

[136] Goodridge JP, Mahmood S, Zhu H, Gaidarova S, Blum R, Bjordahl R, Cichocki F, Chu H-y, Bonello G, Lee T, Groff B, Meza M, Walcheck B, Malmberg K-J, Miller JS, Kaufman DS, Valamehr B (2019). FT596: translation of first-of-kind multi-antigen targeted off-the-shelf CAR-NK cell with engineered persistence for the treatment of B cell malignancies. Blood.

[137] Liu E, Marin D, Banerjee P, Macapinlac HA, Thompson P, Basar R, Kerbauy LN, Overman B, Thall P, Kaplan M, Nandivada V, Kaur I, Cortes AN, Cao K, Daher M, Hosing C, Cohen EN, Kebriaei P, Mehta R, Neelapu S, Nieto Y, Wang M, Wierda W, Keating M, Champlin R, Shpall EJ, Rezvani K (2020). Use of CAR-transduced natural killer cells in CD19-positive lymphoid tumors. N Engl J Med.

